# Radiation Induced Metabolic Alterations Associate With Tumor Aggressiveness and Poor Outcome in Glioblastoma

**DOI:** 10.3389/fonc.2020.00535

**Published:** 2020-05-05

**Authors:** Kshama Gupta, Ivan Vuckovic, Song Zhang, Yuning Xiong, Brett L. Carlson, Joshua Jacobs, Ian Olson, Xuan-Mai Petterson, Slobodan I. Macura, Jann Sarkaria, Terry C. Burns

**Affiliations:** ^1^Department of Neurologic Surgery, Mayo Clinic, Rochester, MN, United States; ^2^Metabolomics Core Mayo Clinic, Rochester, MN, United States; ^3^Department of Radiation Oncology, Mayo Clinic, Rochester, MN, United States; ^4^Department of Biochemistry and Molecular Biology, Mayo Clinic, Rochester, MN, United States

**Keywords:** radiation therapy (RT), glioblastoma (GBM), recurrence, tumor microenvironment (TME), metabolomics

## Abstract

Glioblastoma (GBM) is uniformly fatal with a 1-year median survival, despite best available treatment, including radiotherapy (RT). Impacts of prior RT on tumor recurrence are poorly understood but may increase tumor aggressiveness. Metabolic changes have been investigated in radiation-induced brain injury; however, the tumor-promoting effect following prior radiation is lacking. Since RT is vital to GBM management, we quantified tumor-promoting effects of prior RT on patient-derived intracranial GBM xenografts and characterized metabolic alterations associated with the protumorigenic microenvironment. Human xenografts (GBM143) were implanted into nude mice 24 hrs following 20 Gy cranial radiation vs. sham animals. Tumors in pre-radiated mice were more proliferative and more infiltrative, yielding faster mortality (*p* < 0.0001). Histologic evaluation of tumor associated macrophage/microglia (TAMs) revealed cells with a more fully activated ameboid morphology in pre-radiated animals. Microdialyzates from radiated brain at the margin of tumor infiltration contralateral to the site of implantation were analyzed by unsupervised liquid chromatography-mass spectrometry (LC-MS). In pre-radiated animals, metabolites known to be associated with tumor progression (i.e., modified nucleotides and polyols) were identified. Whole-tissue metabolomic analysis of pre-radiated brain microenvironment for metabolic alterations in a separate cohort of nude mice using ^1^H-NMR revealed a significant decrease in levels of antioxidants (glutathione (GSH) and ascorbate (ASC)), NAD^+^, Tricarboxylic acid cycle (TCA) intermediates, and rise in energy carriers (ATP, GTP). GSH and ASC showed highest Variable Importance on Projection prediction (VIPpred) (1.65) in Orthogonal Partial least square Discriminant Analysis (OPLS-DA); Ascorbate catabolism was identified by GC-MS. To assess longevity of radiation effects, we compared survival with implantation occurring 2 months vs. 24 hrs following radiation, finding worse survival in animals implanted at 2 months. These radiation-induced alterations are consistent with a chronic disease-like microenvironment characterized by reduced levels of antioxidants and NAD^+^, and elevated extracellular ATP and GTP serving as chemoattractants, promoting cell motility and vesicular secretion with decreased levels of GSH and ASC exacerbating oxidative stress. Taken together, these data suggest IR induces tumor-permissive changes in the microenvironment with metabolomic alterations that may facilitate tumor aggressiveness with important implications for recurrent glioblastoma. Harnessing these metabolomic insights may provide opportunities to attenuate RT-associated aggressiveness of recurrent GBM.

## Introduction

Glioblastoma multiforme (GBM; World Health Organization grade IV) is the most common adult primary brain malignancy (1, 2), accounting for 50% of all gliomas across all age groups (2). Standard treatment includes surgical resection, radiation therapy (RT), and chemotherapy; however, the overall 5-years survival rate is <10% with mortality approaching 100% (3, 4) is unfavorable prognosis may be due to the high propensity of tumor recurrence, with many recurring within 1 year, and 90% of these tumors forming within the prior RT field (5–7).

Radiation-induced changes in the brain and tumor microenvironment (TME) injury results in molecular, cellular, and functional changes that can facilitate tumor aggressiveness upon recurrence (8). Such changes include decreased vascularity, innate immune activation, and altered pharmacokinetics, pharmacodynamics, and therapeutic efficacy of chemotherapy agents (9–12). Additionally, irradiation (IR) generated reactive oxygen and nitrogen species (ROS/RNS) play havoc with cellular proteins, DNA, and phospholipid membrane (13). Mitochondria exposed to radiation produce increased ROS that may contribute to RT-induced cell senescence (14–16).

Tumor cell metabolism is strikingly different from that of normal cells with a shift from energy-producing pathways to those generating macromolecules necessary for proliferation and tumor growth. Through a tricarboxylic acid cycle (TCA), healthy cells metabolize glucose and produce carbon dioxide within an oxygen-rich environment, which efficiently produces a large quantity of adenosine triphosphate (ATP) (17). In hypoxic environments, these cells produce large quantities of lactic acid by anaerobic glycolysis. Conversely, in aerobic conditions, tumor cells rely on glycolysis for energy production (18), resulting in elevated rates of glucose uptake and increase lactate production (19). Lactate production during active tumor growth alters the tumor microenvironment by promoting acidosis, serving as a metabolic cancer cell fuel source, and inducing immunosuppression. RT may also have immunosuppressive effects leading to increased tumor aggressiveness, with associated increases in proliferation and infiltration (20), which may be exacerbated by prior RT.

Metabolic alterations may be pro-tumorigenic, promoting glioma initiation and progression (21–25). RT-induced metabolic changes in GBM depend on tumor volume, location, and dose-time regime of RT-administration, all of which can vary treatment response (8, 26–31). While differential metabolism of glioma tumor cells can be targeted for regression of tumor growth, understanding the impact of radiation-induced metabolic alterations in GBM microenvironment can provide new avenues to maximize long term benefits of RT in GBM care. The major objective of this study is to investigate the interactions between irradiation, tumor aggressiveness, and the associated metabolic changes in the TME. We here evaluate the tumor-promoting effects of prior RT on patient-derived intracranial GBM xenograft in mice and characterize the metabolic alterations associated with the pro-tumorigenic stromal microenvironment.

## Materials and Methods

### Ethics Statement on Mice

Six to 8 weeks old female heterozygous Hsd: Athymic Nude-Foxn1nu/Foxn1^+^ mice were purchased from Envigo (Indianapolis, IN). Six to 8-weeks-old male C57BL/6J mice were purchased from Jackson Laboratories (Bar Harbor, ME). Mice were housed at the Mayo Clinic animal care facility, which is accredited by the Association for Assessment and Accreditation of Laboratory and Animal Care International (AAALACI). Aging was induced in two separate cohorts of C57BL/6J mice [fed with regular diet or high-fat diet (D12492, Research diets)] by keeping them in-housed for 24 months (24 mo) at the Mayo Clinic animal care facility, i.e., a small cohort of 5 mice, 2 months old was maintained for 22 months fed throughout on regular diet to obtain an aged mice group (24 mo), and, another cohort of 5 mice (2 months old) was fed on high-fat-diet (HFD) to induce obesity and continued on HFD for 22 months to obtain an aged-obese mice group (24 mo). All animal procedures were performed with proper animal handling, adhering to the National Institutes of Health (NIH) guidelines and protocols approved by the Institutional Animal Care and Use Committee (IACUC) at Mayo Clinic, Rochester.

### Cranial Irradiation of Mice

Cranial irradiation was administered using the X-RAD SmART irradiator (Precision X-ray, North Branford, CT), which uses a cone beam computed tomography (CBCT) for accurate target localization. The stereotactic coordinates were determined from the target-set on CBCT using the first scan for each mouse within all groups (values ranged between *x* = 0.25 to 0.35, *y* = −3.8 to −4.0, and *z* = −5.8 to −5.95, depending on mice and strain-type). Whole brain RT was performed as described (32), using parallel opposed lateral beams with 10 mm square collimator. Radiation treatments included 10 Gy or 20 Gy single dose (20 Gy) administration, or 4 Gy × 10 dose-fractionation. Control group mice were handled similarly as the treated, but with no radiation dose administered (0 Gy).

### Intracranial Injections in Mice

**Figure 1:**
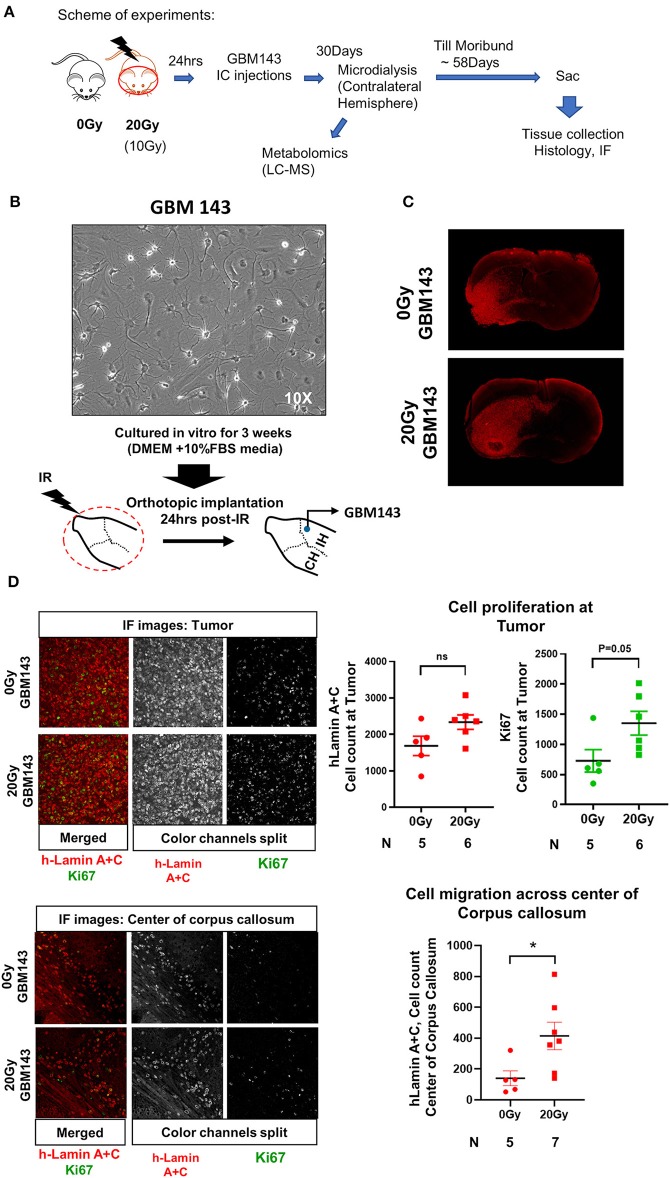
**(A)** Scheme of experiments. **(B)** Representative image (at 10X, transmitted light microscopy) of GBM143 xenograft line cultured for 3 weeks *in vitro* in media indicated; cells were collected and orthotopically implanted into cranially irradiated mice 24 hrs post-irradiation (IR). **(C)** Representative Immunofluorescence (IF) images (at 4X, tiling) for hLamin A+C staining from 0 Gy and 20 Gy-IR mice coronal sections to assess tumor growth and invasion. **(D)** Top: Representative images (at 20X) show IF staining at tumor; and the dot-plot of single cell count for hLamin A+C and Ki67 staining. Bottom: Representative images (at 20X) show IF staining at center of corpus callosum, and dot-plot of single cell count for hLamin A+C. IH, ipsilateral hemisphere; CH, contralateral hemisphere; IR, irradiation. Statistical significance is represented as ^*^p < 0.05.

Intracranial (IC) injections in athymic nude mice were performed as previously described (33). Briefly, pre-established human GBM xenograft line, GBM143 cells were obtained from flank tumors and cultured *in vitro* in Dulbecco's Modified Eagle Medium (DMEM, Gibco™ 41966029) media having 10% Fetal bovine serum (FBS) and antibiotics (penicillin-streptomycin), for 3 weeks (34, 35). Representative images for GBM143 cell growth were acquired in transmitted light, using EVOS^®^ FL Cell Imaging System, Thermo Fisher Scientific (Figure 1B, Figure S1A). These cells were dissociated using TryplE (Cat# 12563011, Thermo Scientific) and resuspended in PBS at a concentration of 100,000 cells per μl (with injection volume 3 μl/mouse). Mice were anesthetized using Ketamine: Xylazine mixture (100 mg/kg Ketamine and 10 mg/kg Xylazine) injected intraperitoneally (IP) with a 0.5cc syringe. The surgical procedure involved the following steps: disinfecting mice head with Betadine, lubricating the eyes with artificial tears, making a 1 cm midline incision extending from just behind the eyes to the level of the ears using sterile scalpel while applying pressure to have the incision open. Using a cotton swab, the skull was cleared to have the bregma exposed, a point 1 mm anterior and 2 mm lateral from bregma was identified and drilled through the skull using an 8bit Dremel drill. For stereotactic injection, Hamilton syringe with a 26G needle assembly was cleaned thoroughly, fixed on the injection jig, and 3 μl of cell suspension drawn into it. Injection jig was sterilized by wiping with STERIS Spor-Klenz and draping it with a sterile towel. The mouse having its skull drilled was placed on the jig and fixed using a front teeth hook at mouthpiece and ear pins. Using the stereotactic controls, the needle was inserted 3 mm deep into the brain and, cell suspension having 300,000 cells/3 μl was injected at a rate of 1 μL/min for over 3 min using the syringe pump. The needle was maintained as inserted in place inside the skull for additional 1 min, and then drawn out gently using the stereotactic controls. The hole drilled in mouse-skull at site of tumor cell implantation was sealed using bone cement, and the wound sutured with 4-0 vicryl with rb-1 needle (Ethicon J304H). Triple antibiotic was applied to the incision and stitches to prevent infection, and the mouse was left in the warm cage to recover from anesthesia. Water was supplemented with children's ibuprofen starting 24 hrs prior to the procedure and continued for 48 hrs post-surgery. The scheme of experiments involving IC injections is illustrated in Figures 1A,B and Figure S1F.

### Histology and Immunofluorescence

Athymic nude mice injected with the established PDX line, GBM143, were euthanized using isoflurane overdose at day of moribund (i.e., after 58 days of tumor cell-implantation). PBS cardiac perfusion was performed prior to termination under fully anesthetized conditions to remove the circulating peripheral leukocytes from the brain. Brains were extracted, fixed in 10% buffered formalin for 24 hrs, paraffin embedded, and 5 μm coronal sections were obtained (slicing strategy explained in Figure S1B). All processing after fixation was performed at Mayo Clinic Histology core, Scottsdale. For histologic analysis, slides were stained with hematoxylin and eosin (H&E) and visualized by bright field microscopy at 4X microscopic magnification using Leica DMI-6000B [software: Leica Application Suite X (Leica Microsystems, Wetzlar, Germany)]. Percent positive H&E stained area was assessed as illustrated in Figure S1C, to estimate relative tumor burden between the samples.

H&E stained sections were reviewed to identify appropriate tumor bearing regions and respective unstained slides processed for immunofluorescence (IF) staining with human-Lamin A+C and Ki67 antibodies using standard procedure. Briefly, slides were deparaffinized in xylene and rehydrated by washing (3 min each) in serially diluted ethanol from 100, 95, 75, 50%, and then distilled H_2_O. Antigen retrieval was performed using pre-warmed 9.8 mM Sodium citrate buffer (pH 6.0, with 0.05%Tween 20) for 30 min in hot steamer. Slides were rinsed in distilled H_2_O and PBS, blocked in blocking solution (10% Normal goat serum and 1% BSA in PBS), and stained with primary antibody (diluted in blocking solution, 1:300) overnight in humidified chamber at 4°C. The slides were washed in PBS (3 × 5min), stained with secondary antibody (diluted in blocking solution, 1:300) for 2 hrs at room temperature, washed, and mounted with ProLong Gold reagent having DAPI (P36935, Life Technologies). Images were acquired at 4X microscopic magnification and tiling was done using Leica DMI-6000B (software: Leica Application Suite X).

#### Image Analysis

All IF stained slides were quantified and scored for single cell count in a defined region with x-y coordinates approximated at tumor center (for h-Lamin A+C, and Ki67) or at center of corpus callosum (for h-Lamin A+C), respectively, using Image J (36, 37) and Cell Profiler 2.2.0 (Broad Institute of Harvard and MIT) (38). Briefly, for single cell counting, an IF image obtained was imported into Image J, threshold was set, channels split, and image in relevant single channel was selected and converted to black and white (BW). An area template having fixed size was generated to define a contained region at tumor or at the center of corpus callosum, respectively, maintaining consistency between different sample slides. This defined area selectively masked was overlaid and appropriately positioned in the BW image, and all background cells out of the masked region were eliminated. The resultant image was transferred to Cell Profiler 2.2.0 software (Broad Institute of Harvard and MIT) (38), the masked region was cropped and used as input; the pipeline for single cell counting was run to detect nuclei and quantify cells within this defined region.

To evaluate microglial activation, slides were stained for Iba-1 using standard procedure for IF. Images represented with 20X magnification were acquired on Leica DMI-6000B (software: Leica Application Suite X) and 40X magnification on Zeiss Axio Observer Z.1 (Software: Zen 2.3 SP1, Jena, Germany). Microglial morphology was assessed using ImageJ (36, 37). Antibodies used: Rabbit monoclonal Anti- h-Lamin A+C [EPR4100] (Cat# Ab108595, Abcam, Cambridge, United Kingdom); Rat monoclonal Ki67 (SolA15) (Cat #14-5698-82, eBioscience Invitrogen, Waltham, MA); Rabbit monoclonal Anti-Iba-1 (Cat# 019-19741, Wako). Secondary antibodies from Jackson ImmunoResearch Laboratories, Inc. (West Grove, PA) included polyclonal affinity-pure whole IgG: Cy3-Goat Anti-Rabbit IgG (H+L) (code: 111-165-003) and Cy5-Goat Anti-Rat IgG (H+L) (code: 112-175-143).

### Microdialysis

To evaluate changes in the extracellular milieu of radiated brain, a small group of mice (3 mice per group) from 0 Gy and 20 Gy single-dose irradiated mice cohorts injected with GBM143 24 hrs post-IR, were microdialyzed on their contralateral hemisphere (non-tumor bearing side) at day 30 from tumor cell injection (scheme of experiment in Figure 1A). The microdialysis set-up and surgical procedure was followed as described from the facility of Dr. Doo-Sup Choi, at Mayo Clinic, Rochester, Minnesota (39). Briefly, the mice were housed singly for 2 hrs in the microdialysis room to acclimatize, and then anesthetized using Ketamine:Xylazine mixture. Survival surgery was performed on a rotating platform with stereotactic guidance under sterile conditions. A microdialysis probe with a 2.0 mm cellulose membrane (Brain Microdialysis, CX-I Series, Eicom, Kyoto, Japan; MW cut off: 50,000 Da) was inserted at a point 1 mm anterior and 2 mm lateral from bregma on the contralateral hemisphere and secured to the guide cannula. The probe was connected to a microsyringe pump (Eicom, Kyoto, Japan), which delivered Ringer's solution (145 mM NaCl, 2.7 mM KCl, 1.2 mM CaCl_2_, 1.0 mM MgCl_2_, pH 7.4) at a 1.0 μl/min flow rate. The samples were collected in 0.2 ml collection tubes maintained at 4°C for 3.5 hrs, and then immediately frozen and stored at −80°C until analyzed.

### Metabolomics

To assess the radiation induced metabolic alterations in the pre-radiated mice brain, Proton Nuclear magnetic resonance spectroscopy (^1^H-NMR) and Gas Chromatography- Mass Spectrometry (GC-MS) based metabolomics was performed on whole tissue extracts obtained from non-tumor bearing brain samples of two independent strains of mice: Athymic nudes and C57BL/6. The experimental design with mice groups included for each strain is illustrated in Figure S2A.

#### Proton Nuclear Magnetic Resonance Spectroscopy (^1^H-NMR)

Athymic nude mice, 0 Gy-control, and 20 Gy single-dose irradiated (10 mice per group) were sacrificed and immediately frozen in liquid nitrogen. Brian tissues were collected on dry ice and pulverized in liquid nitrogen. The pulverized mouse brain tissue (~55–60 mg) was homogenized and extracted with 300 μl of ice-cold 0.6 M perchloric acid (HClO_4_) solution. Samples tubes were vortexed, centrifuged at 10,000 g for 10 min at 4°C, and supernatants collected (40). The extraction procedure was repeated on the pellets (with ~150 μL HClO_4_) and supernatant obtained from two rounds of extraction were combined and neutralized with 140 μl of 2M potassium bicarbonate (KHCO_3_). In 400 μL aliquot of neutralized extract, 100 μL of 0.1M phosphate buffer, and 50 μL of 1 mM TSP-*d*_4_ in D_2_O were added. Samples were vortexed for 20 s and transferred to 5 mm NMR tubes. The NMR signal was acquired on Bruker AVANCE III 600 MHz instrument (Bruker, Billerica, USA). ^1^H-NMR spectra were recorded using 1D NOESY pulse sequence with presaturation (noesygppr1d) under the following conditions: 90-degree pulse for excitation, acquisition time 3.90 s, and relaxation delay 5 s. All spectra were acquired with 256 scans at room temperature (298 K) with 64k data points and 8,417 Hz (14 ppm) spectral width. The recorded ^1^H-NMR spectra were phase and baseline corrected using TopSpin 3.5 software (Bruker, Billerica, MA). The spectra were then processed using Chenomx NMR Suite 8.3 software (Chenomx Inc., Edmonton, Canada). The compounds were identified by comparing spectra to database Chenomx 600 MHz Version 10 (Chenomx Inc., Edmonton, Canada) and literature data (40–46). Quantification was based on an internal standard (TSP-*d*_4_) peak integral. The metabolite concentrations were exported as μM in NMR sample and recalculated as μmol/g of wet tissue.

#### Gas Chromatography–Mass Spectrometry (GC-MS)

For GC-MS analysis, 70 μl neutralized brain extracts (~6.4 mg of tissue wet weight) from athymic nudes were obtained using perchloric acid extraction method with 2M KHCO_3_ based neutralization as described for ^1^H-NMR, centrifuged at 10,000 g for 10 min, and cleared supernatant collected in fresh 1.5 ml eppendorf tubes. These samples were completely dried in a SpeedVac concentrator run overnight. They were subsequently methoximated using 10 μL MOX^TM^ reagent (Cat# TS-45950, ThermoScientific, Waltham, MA) at 30°C for 90 min and then derivatized using 40 μL of N-methyl-N-trimethylsilyl trifluoroacetamide with 1% trimethylchlorosilane (MSTFA+1% TMCS: Cat# TS48915, ThermoScientific, Waltham, MA) at 37°C for 30 min. Metabolite levels were determined using GC-MS (Hewlett-Packard, HP 5980B) with DB5-MS column. GC-MS spectra were deconvoluted using AMDIS software (NIST, Gaithersburg, MD) and SpectConnect software (Georgia Tech, Atlanta, GA, USA) was used to create metabolite peaks matrix. The Agilent Fiehn GC/MS Metabolomics RTL Library (Agilent, Santa Clara, CA) was used for metabolite identification. Ion count peak area was used for analysis of the relative abundance of the metabolites (47).

Similar to above, whole brain extracts using perchloric acid method were also prepared from a cohort of C57BL/6 mice and evaluated by ^1^H-NMR and GC-MS. C57BL/6 mice included in the study were divided into five groups (with 4–5 mice per group) as follows: control (0 Gy), 20 Gy single-dose irradiated, 4 Gy × 10 fractionation-dose irradiated, aged (24 mo), and aged-obese (24 mo) (scheme included in Figure S2A).

#### Data Analysis

Multivariate analysis of NMR data was performed using SIMCA 15 software (Sartorius Stedim Biotech, Göttingen, Germany). Principal component analysis (PCA) was used to detect any innate trends and potential outliers within the data. Supervised Partial Least Squares discriminant analysis (PLS-DA) and Orthogonal-Partial least square–discriminate analysis OPLS-DA were performed to obtain additional information including differences in the metabolite composition of groups, variable importance on projection (VIP) values, and regression coefficients. OPLS-DA models were calculated with unit variance scaling and the results were visualized in the form of score plots to show the group clusters. The VIP values and regression coefficients were calculated to identify the most important molecular variables for the clustering of specific groups. Non-parametric Wilcoxon rank sum test and Student *T*-test were performed to determine the statistically significant differences between the groups.

### Survival Curves

Athymic nudes, grouped as control (non-irradiated, 0 Gy) and irradiated with 20 Gy single dose, were divided into two study cohorts: (1) Short-term IR: where 5 mice from each group were injected with GBM143 cells after short-term prior IR-exposure of 24 hrs, and (2) Long-term IR: where 5 mice from each group were maintained for 2 months post-irradiation and then injected with GBM143 cells. Survival time (in days) for each mouse was recorded until 70 days post tumor cell injection. The overall survival was calculated by Kaplan-Meier method and log-rank test was used to compare the survival curves (48). Experimental design illustrated in Figure S1F.

### Statistical Representation

The difference between specific metabolites or a parameter measured across two groups was estimated for *p*-value, as indicated. Graphs were plotted using software(s): GraphPad Prism 8.2.0 (GraphPad, San Diego, CA), Heatmapper (Wishart Research Group, University of Alberta and Genome Canada) (49) and Microsoft Office Excel. Statistical significance is represented as *p*-values: ^*^p < 0.05; ^**^p < 0.01; ^***^p < 0.001, ^****^p < 0.0001.

## Results

### Effect of Radiation on Tumor Growth, Proliferation and Migration

Mice were cranially irradiated with either 20 Gy (single dose) or 0 Gy (control), and tissues were collected at moribund to be evaluated with histology for tumor growth. A small cohort of mice radiated with 10 Gy (single dose) and injected with GBM143 line was also compared with the 0 Gy and 20 Gy cohorts for relative tumor burden using haematoxylin and eosin (H&E) staining. No difference in tumor size was observed between 0 Gy and 10 Gy; however, 20 Gy irradiated samples had significantly higher percent of section area positive for tumor, indicated by H&E (~15% positive H&E for 0 Gy and 10 Gy, and 27% for 20 Gy, with *p*-value of 0.033 between 0 Gy and 20 Gy), indicating an overall faster rate of tumor growth (Figure S1D). Thus, 10 Gy cohort was not pursued for further evaluation. Sections from 0 Gy and 20 Gy were analyzed for tumor growth and proliferation using human-Lamin A+C and Ki67 staining. There was observable difference between tumor size of 0 Gy and 20 Gy radiated mice based on h-Lamin A+C staining at the tumor. Also, more cells positively stained for h-Lamin A+C were present at the corpus callosum of 20 Gy mice (Figure 1C). Quantitative analysis performed by counting both h-Lamin A+C and Ki67 within the tumor to evaluate proliferation revealed higher trend of both the stains in 20 Gy, with h-Lamin A+C. The h-lamin A+C was however not significant, due to high-density tumor region evaluated for both 0 Gy and 20 Gy; but showed near to significant difference in Ki67 positive cells stained in that area (*p* = 0.05), indicating higher proliferative potential in tumors that were obtained from 20 Gy-pre-irradiated mice brain. Similarly, h-Lamin A+C was assessed in the midline corpus callosum to evaluate cell migration toward the contralateral hemisphere, as illustrated in Figure S1E. The h-Lamin A+C staining in 20 Gy was significantly higher with a *p*-value of 0.03, compared to 0 Gy mice in the midline corpus callosum, suggesting a higher number of cells migrating toward the contralateral hemisphere (Figure 1D).

### Metabolomics

#### Microdialysis

To assess for radiation-induced changes in the extracellular milieu, a pilot experiment with intracranial microdialysis (in the contralateral hemisphere) was performed in a small cohort of athymic nude mice (*n* = 3), involving groups 0 Gy and 20 Gy, at day 30 after GBM143 injection and microdialysates were analyzed for untargeted liquid metabolic profiling using LC-MS (method described in Supplementary Materials 1). Principal component analysis could separate the groups 0 and 20 Gy, indicating metabolic changes in effect of irradiation. A trend toward elevated levels of metabolites relevant to cancer progression was observed in the 20 Gy mice, including modified nucleotides (N6-methyladenosine, pseudouridine), polyol (myo-inositol, quebrachitol) detected in the 20 Gy (Supplementary Excel Sheet 3). However, there were very limited identifiable metabolites with a total of <60 due to low sample volume obtained after a 3.5 hrs microdialysis run at a rate of 1 ul/min (Supplementary Excel Sheet 3). Moreover, due to technical challenges involved with keeping ≥4 mice per group in microdialysis and the limited volume of microdialysates collected for evaluation, significant conclusions could not be made. We therefore utilized a whole tissue metabolomics approach in non-tumor bearing mice to evaluate the metabolic changes post-irradiation.

#### Proton Nuclear Magnetic Resonance (^1^H-NMR) Spectroscopic Analysis

We sought to identify the radiation induced metabolic alterations in the brain stroma associated with the observed outcome of higher tumor growth and proliferation in 20 Gy mice. To achieve this, whole brain metabolomics was performed in two separate mouse strains, athymic nude mice and C57BL/6 mice, as described in methods. Athymic nude mice were included since the tumor study described above was performed with human-PDX line in athymic nudes; C57BL/6 mice were included to eliminate strain dependence and to avoid potential confounding effects of immunodeficient mice. A cohort of aged-C57BL/6 mice (24 mo) with and without diet-induced-obesity was analyzed to assess whether or not the radiation-induced metabolic changes in the brain were similar to those induced by aging or obesity-induced senescence. A small group of C57BL/6 mice were administered a fractionated dose of 4 Gy × 10 for comparative analysis.

**Figure 2:**
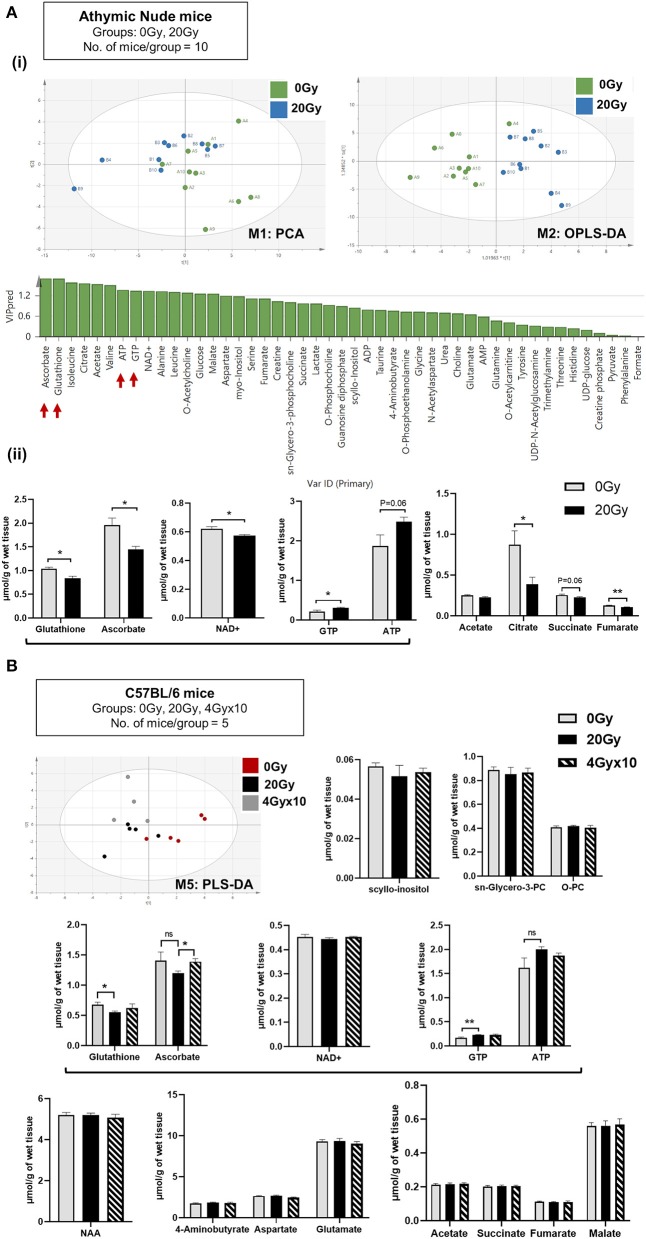
Metabolomics of pre-radiated brain using ^1^H-NMR: **(A)** (i) Principal component analysis (PCA) between athymic nude mice groups, 0 Gy and 20 Gy. Orthogonal Partial Least Squares-Discriminant Analysis (OPLS-DA) shows further separation of 20 Gy mice group from non-irradiated control based on differences in the metabolite composition of groups with predicted-variable importance in the projection values shown in graph below. (ii) The graphs show, significantly altered metabolites between the 0 Gy vs. 20 Gy. **(B)** Multivariate analysis of NMR data performed using SIMCA 15 software (Sartorius Stedim Biotech, Göttingen, German) for cohort of C57BL/6 mice, having groups as indicated. Supervised Partial Least Squares discriminant analysis (PLS-DA) performed shows, separation of all three groups. Bar graphs show metabolites most significantly altered between groups. Additional graphs for metabolic variants observed in C57BL/6 mice are included in Figure S3. Statistical significance is represented as ^*^p < 0.05; ^**^p < 0.01.

Data ^1^H-NMR spectroscopic analysis revealed clear separation of 0 and 20 Gy mice cohorts from athymic nude mice, using PCA (Figure 2Ai). Supervised OPLS-DA further separated the two groups based on metabolite composition differences with predicted-variable importance in the projection (VIP) values shown. The most important molecular variables for clustering of specific groups include glutathione (GSH) and ascorbate (ASC) having VIPpred 1.65, along with differences in ATP and GTP levels as potentially distinguishing characteristics (Figure 2Ai). After IR, a significant reduction of GSH, ASC, and NAD^+^ levels were observed, along with increases in ATP and GTP. Additionally, an overall reduced trend in TCA intermediates was observed in 20 Gy (Figure 2Aii). The multivariate analysis of NMR data performed using SIMCA 15 software for C57BL/6 mice demonstrated separation of groups: Aged 24 mo, Aged-Obese 24 mo, Control (0 Gy), 20 Gy single-dose cranially irradiated, and 4 Gy × 10 cranial IR-fractionated. Supervised PLS-DA showed separation of 0 Gy from irradiated mice groups, 20 Gy and 4 Gy × 10 (Figure 2B) and all five groups (Figure S2B). Specifically, the aged-groups (aged: 24 mo and aged-obese: 24 mo) were separated into a different component compared to the 0, 20, and 4 Gy × 10 groups (Figure S2B). There was a better separation of groups shown in model: M4 (aged, 0 and 20 Gy) as compared to those shown in model:M5 (0, 20, and 4 Gy × 10) (Figure S2B, Table S1 for model parameters). Comparing all irradiated mice (IR group: 20 and 4 Gy × 10 analyzed together) with 0 Gy using PLS-DA and OPLS-DA showed significant group separation. The VIP-total and VIPpred value estimation indicated the metabolites most relevant to this group separation, which included GTP, ATP, GSH, and ASC (Figures S2Bi,ii).

The relative abundance of metabolites identified post-IR for 20 Gy single dose from ^1^H-NMR for C57BL/6 mice showed reduction in GSH and ASC levels and an increase in ATP and GTP. No significant difference was observed between 20 and 4 Gy × 10 (Figure 2B). To assess how metabolomic profile of the radiated brain (at doses 20 Gy, and, 4 Gy × 10) was different from age-related brain metabolomic profile, C57BL/6 aging-mice cohorts (24 mo) were evaluated for significantly altered metabolites in comparison to irradiated and control mice. Alterations specific to the aged-group involved increased levels of scyllo-inositol and sn-glycero-3-phosphocholine with concomitant reduction in O-phosphocholine. Other metabolites reduced significantly in aged-mice were NAA (N-acetyl aspartate), neurotransmitters, and intermediates of TCA cycle (Figure S3) (50–54). List of metabolites detected for athymic nude mice and C57BL/6 by ^1^H-NMR are included in Supplementary Excel sheet 1.

#### Gas Chromatography-Mass Spectrometry (GC-MS)

**Figure 3:**
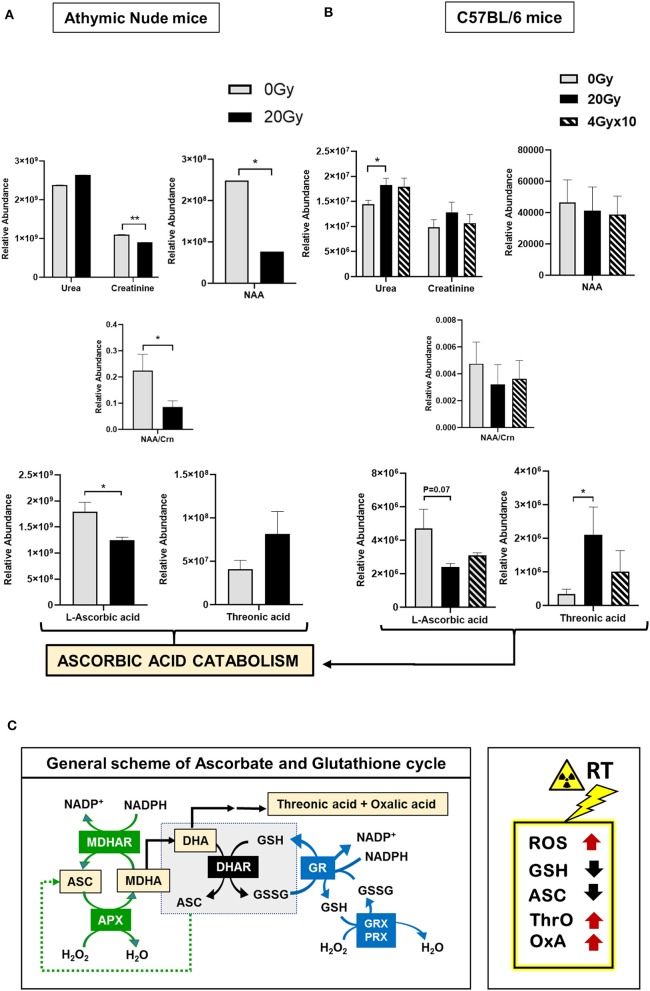
Metabolomics of pre-radiated brain using GC-MS: **(A)** The graphs show significantly altered metabolites between athymic nude mice groups, 0 Gy and 20 Gy. **(B)** The graphs show significantly altered metabolites between C57BL/6 mice grouped 0, 20, and 4 Gy × 10. Additional graphs for metabolic variants in C57BL/6 mice groups are in Figure S4. Heatmaps for GC-MS are included in Figure S2C. **(C)** General scheme for ascorbate (ASC) and glutathione (GSH) cycle in clearance of reactive oxygen species (ROS). Intermediates of ascorbic acid catabolism are represented in orange boxes. Reactions in green show ASC-dependent peroxide metabolism; reactions in the central gray box show GSH-dependent regeneration of ASC; and reactions in red show GSH-dependent peroxide metabolism. Box on the right illustrates the expected metabolic alterations upon irradiation, which include increases in levels of ROS, and utilization of GSH and ASC, with concomitant increase in by-products of ASC catabolism, Threonic acid (ThrO), and Oxalic acid (OxA). Key to metabolic cycle illustrated: ASC, Ascorbate; MDHA, Monodehydroascorbate; MDHAR, Monodehydroascorbate reductase; APX, ASC peroxidase; GR, GSH reductase; GRX, Glutaredoxin; PRX, Peroxiredoxin; ThrO, L-threonic acid; OxA, oxalic acid; DHA, Dehydroascorbic acid; GSH, Glutathione reduced; GSSG, Glutathione, oxidized; NADP^+^, Nicotinamide adenine dinucleotide phosphate. Statistical significance is represented as ^*^p < 0.05; ^**^p < 0.01.

Lysates processed for ^1^H-NMR were further evaluated using GC-MS. The heatmap for relative abundance of metabolites (i.e., normalized total peak area of a metabolite per mice), between athymic nude mice, 0 Gy and 20 Gy, is illustrated in Figure S2Ci. While there was internal variation observed within these groups, only a few significantly altered metabolites in 20 Gy were identified, which included an increased trend in urea and a reduction in levels of creatinine (Crn), N-acetyl aspartate (NAA), and NAA/Crn ratio post-irradiation. Importantly, ascorbic acid was significantly reduced in 20 Gy and threonic acid was increased, reflecting ascorbic acid catabolism (Figure 3A). The heatmap for relative abundance of metabolites averaged for each group of C57BL6 mice is included in Figure S2Cii. The significantly altered metabolites between control (0 Gy) and irradiated groups (20 Gy and 40 Gy × 10) involved increased levels in urea but no change in Crn, NAA, and NAA/Crn ratio. However, there was significant reduction in levels of ascorbic acid with concomitant rise in threonic acid observed post-irradiation, alike observed for the athymic nudes (Figure 3B). Collectively, the results of ^1^H-NMR and GC-MS indicate involvement of ROS clearance with active utilization of GSH and ASC as antioxidants. Scheme for ASC and GSH cycle in clearance of ROS and the role of GSH in regeneration of ASC is illustrated, along with intermediates of ascorbic acid catabolism, in Figure 3C. Expected metabolic alterations upon irradiation involve an increase in levels of ROS, utilization and reduction in GSH and ASC, with concomitant increase in by-products of ASC catabolism, threonic acid (ThrO), and Oxalic acid (OxA) (Figure 3C).

Other metabolites contributing to the separation of the groups in C57BL6 mice, and their relative assessment with aged mice groups are shown in Figure S4. The heatmap showed distinct metabolomic signatures for aging from that of irradiation (Figure S2Cii). At individual metabolite levels, no significant difference was observed for cholesterol in aged-Obese mice, which could be due to high internal variation observed within the group or small cohort size (5 mice/group). However, there was a reduced trend in free fatty acids and overall higher cholesterol, as compared to others. Notable metabolites separating the aged groups from the irradiated involved: increased age-related markers, scyllo-inositol and sn-glycero-phosphocholine, and reduction in fumaric, succinic acids, and metabolic intermediates of glycolysis and TCA cycle (52–54). Metabolic variations common to both aged and radiated mice cohorts included a rise in threonic acid, oxalic acid, D-allose, and myo-inositol. Additionally, there was a slightly higher trend in urea and Crn; however, this was not significant for either aged or irradiated mice groups (Figure S4). List of metabolites detected by GC-MS are included in Supplementary Excel sheet 2.

### Immunostaining for Microglia With Iba-1

**Figure 4:**
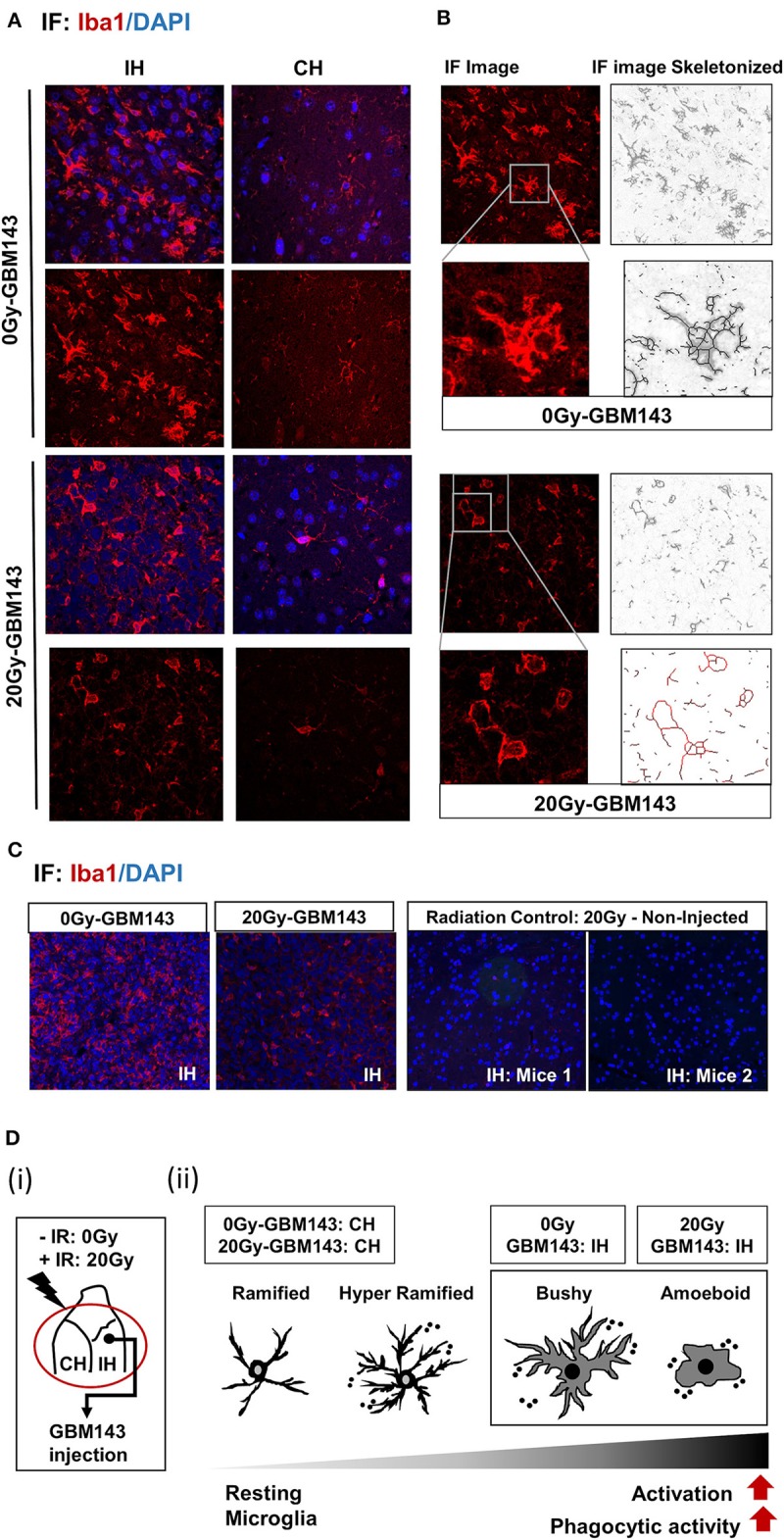
Immunostaining for microglia, with Iba-1: **(A)** Immunofluorescence (IF) Images for microglial staining (Iba1/DAPI) and morphology (at 20X), in ipsilateral (IH) and contralateral (CH) hemispheres of 0 Gy and 20 Gy mice injected with GBM143 PDX line. **(B)** the IF images skeletonized using Image J software to assess microglial morphology. **(C)** the microglial staining in ipsilateral hemispheres of 0 Gy-GBM143, and 2 Gy-GBM143 compared with that of ipsilateral hemispheres of two separate mice cranially irradiated with 20 Gy-single dose; however, not injected with any human-GBM PDX line. **(D)** (i) Site of GBM143 injection at IH of mice having received ± cranial irradiation (ii) Stages of microglial activation observed in experimental setting.

To evaluate the status of inflammation in the radiated brain and tumor microenvironment in response to RT, immunostaining for Iba-1 was performed for microglia in coronal slices from mice cranially irradiated (0 Gy or 20 Gy), and injected 24 hrs post-IR with GBM143 PDX line (Figure 4). Microglial morphology was assessed in ipsilateral (IH) and contralateral (CH) hemispheres Microglia were observed to be enlarged, bushy, and branched for 0 Gy-GBM143, as opposed to amoeboid for 20 Gy-GBM143, indicating stages of higher activation and higher phagocytic activity for the 20 Gy-GBM143 injected mice (Figures 4A,B). Comparing the microglial staining in ipsilateral hemispheres of 0 Gy and 20 Gy-GBM143 with that of the ipsilateral hemispheres of two separate mice that were cranially irradiated with 20 Gy but not injected with any human-GBM PDX line (radiation controls): showed, negligible Iba1^+^ microglia staining in the brain slices of 20 Gy-IR alone, indicating, the observed microglial activation to be an effect of crosstalk between irradiation and tumor pathogenesis. Figure 4D illustrates their relevance in our experimental setting with maximum microglial activation and phagocytic activity observed in 20 Gy mice.

### Effect of Radiation on GBM Outcome

**Figure 5:**
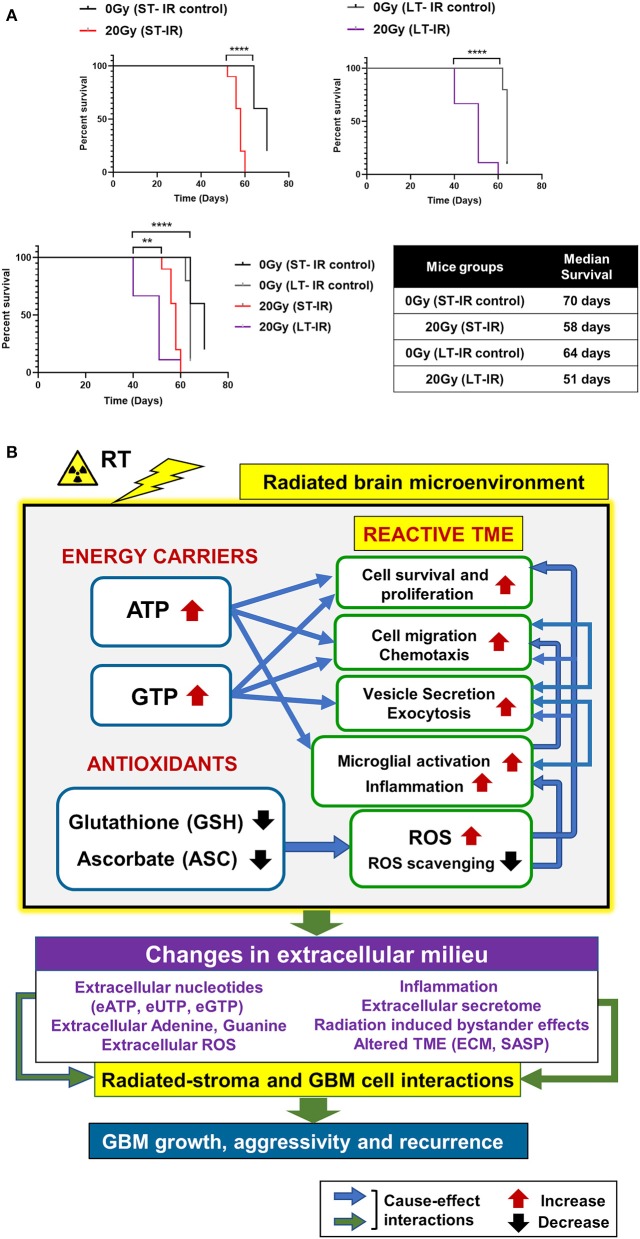
Effects of radiation-associated metabolic alteration on GBM outcome: **(A)** Survival curves: Graphs show difference between survival of irradiated (IR) mice cohorts injected with GBM143 24 hrs post-irradiation (short-term IR, ST-IR) or 2 months post-irradiation (long-term IR, LT-IR). Statistical significance is represented as ^**^p < 0.01, ^****^p < 0.0001. **(B)** Proposed model for radiation associated metabolic alterations and their effects on cell processes and glioblastoma multiforme (GBM) outcome. The box marked with yellow outline (irradiated by RT) shows metabolic changes in the radiated brain stromal microenvironment, with rise in energy carriers ATP and GTP, and reduction in levels of antioxidants, ascorbate and glutathione, and the cellular processes affected by them. With IR, continued and excess rise in levels of energy carriers and expense of antioxidants within stromal cells of the brain can lead to altered extracellular milieu. Pathophysiological changes in the extracellular milieu, which can be immediate or long term caused by the radiation are enlisted in purple box. These alterations would collectively contribute to radiated stroma and GBM cell interactions that are permissive to GBM growth and, aggressive recurrence. Translational relevance of the study and its insights gained from pre-radiated brain microenvironment to prevent secondary and recurrent GBM spread is illustrated in Figure S5.

Effect of radiation-associated metabolic alteration on GBM outcome was assessed by, survival analysis for irradiated mice cohorts, ST-IR and LT-IR, and their respective control groups, injected with GBM143. Significant reduction was seen in the survival of mice after irradiation ST-IR or LT-IR (Figure 5). The combined graph of ST-IR and LT-IR further showed a significant difference is survival of 20 Gy (ST-IR) and 20 Gy (LT-IR) with median survival of 58 and 51 days, respectively.

Collectively, our data demonstrate radiation-induced metabolic alterations, including a rise in energy carriers (ATP and GTP) and reduction in antioxidants (GSH and ASC) associated with tumor promoting cell processes (cell proliferation, migration, and inflammation) and poor GBM outcome. The proposed model is illustrated in Figure 5B, and its translational significance is illustrated in Figure S5.

## Discussion

Radiation therapy (RT) is an indispensable treatment modality for management of majority of cancers, and the standard of care for GBM. While, RT exerts its therapeutic potential by killing the proliferative tumor cells, RT can severely impact the TME by altering the extracellular milieu at molecular and structural levels (8, 26, 55). Radiation induced brain injury is widely documented; however, pro-migratory effects of RT on GBM cells have recently gained attention. Independent groups have reported enhanced human glioma cell migration and invasion in response to radiation dose treatment (56–59). We here evaluated the tumor growth and migratory potential of human-PDX line (GBM143) in the pre-radiated brain microenvironment with purpose to recapitulate the tumor recurrence scenario observed in clinic. As a measure of tumor growth and invasion, we quantified the proliferative cells at tumor and migratory cells crossing the center of corpus callosum and, observed higher cell migration and proliferation of GBM143 PDX line implanted in mice brain pre-radiated with 20 Gy indicating a tumor permissive microenvironment of the brain post-RT (Figure 1D) (8).

Radiation treatment leads to production of ROS, which facilitates tumor cell cytotoxicity in effect of RT. Tumor cells adapt to this oxidative stress through several mechanisms, including metabolic shifts and elevated antioxidant peptide production and intratumoral hypoxia generation (60–62). However, the RT-induced redox state of the non-transformed cells in the tumor stroma and how it may cross-interact with transformed tumor cells to impact tumor growth is less studied. Increased ROS levels in response to IR can be pro-tumorigenic (21, 22).

Metabolomics has emerged as the state-of-the-art approach to identify cancer cell fate (63–69); and metabolic therapy for management of GBM has been discussed (70, 71). We evaluated the metabolic changes in the pre-radiated brain microenvironment in response to 20 Gy-IR and the association with observed tumor aggressivity and inflammatory microglial phenotype. Cell proliferation and migration are a direct function of the cell's energy state (21); therefore, utilizing ^1^H-NMR we quantified energy carriers in the radiated brain stroma and, found elevated levels of ATP and GTP post 20 Gy-IR with reduced levels of antioxidants, glutathione, and ascorbate (Figure 2). Ascorbate and GSH serve as the prime cellular antioxidants. Glutathione can recycle itself and reduced ascorbate (72, 73). Active ASC catabolism with decreased levels of ASC and GSH were observed, which indicate active ROS scavenging. While studies have also shown reduced intracellular redox signaling pathway in response to radiation, which may contribute to radiation induced oxidative stress (74), the depletion of ROS scavengers due to their increased demand would cause further accumulation of intracellular ROS, exacerbating oxidative stress. Chronically high levels of ROS in the TME can facilitate tumor growth (62, 75). Similarly, while ATP and GTP are essential components of cellular homeostasis, a rise in these intracellular nucleotides can cause their export out of the cell through extracellular vesicles, thus elevating their levels in extracellular space (76, 77). Extracellular purinergic nucleotides can affect both stroma and tumor cell processes. Extracellular ATP (eATP) has been implicated in facilitating microglial chemotaxis, inflammation, and several neurological or neuropathological processes (78). Additionally, it can be internalized by tumor cells, increasing their intracellular ATP levels conferring metabolic reprogramming, increased tumor aggressivity, and treatment resistance (79–83). A recent lung cancer study has shown eATP to be involved in epithelial-to-mesenchymal transition, cell migration, and metastasis (84). While the biological functions of extracellular guanosine or eGTP are less studied than adenosine or eATP, their relative concentrations can co-vary, and biological functions of these nucleotides can cross-interact (85, 86). GTP is an essential biomolecule that modulates cell signaling via G-proteins and small GTP-binding proteins to facilitate cell proliferation, cell migration, and vesicle trafficking, and, can modulate metabolism and tumor development (87–94). Exocytosis and vesicle secretion can further facilitate release of purinergic nucleotides, inflammatory molecules, enzymes, and ROS into the extracellular milieu, which collectively can alter the TME to become pro-tumorigenic (75, 83, 95–97). These cause-effect relation between metabolic alterations and their cell physiological processes in radiated brain stroma, are illustrated in Figure 5B.

Microglia are the prime cells of immune surveillance in normal brain and one of the main the cellular components of tumor associated macrophages (TAMs) in the immune microenvironment of GBM (98–101). A persistent activation of microglia is the hallmark of a chronic neuroinflamation. Microglial activation and its M1 polarization state is characteristically exhibited in traumatic brain injury; however, the extent to which M1 vs. M2 polarization states relate to radiation-induced changes in microglia remains unclear (8, 32, 102, 103). Upon inflammatory trigger, microglial activation matures with sequential changes in its morphology, from resting ramified state to hyper-ramified, bushy and highly phagocytic ameboid state (101, 104, 105). A prolonged activation of microglia leads to a vicious circle, where secretion of pro-inflammatory cytokines and other neurotoxic agents (ROS and RNS) leads to further neuronal damage and cell death, which maintains microglial cells in their activated status (103, 106, 107). Extracellular ATP (eATP) can act as a chemoattractant and facilitates microglial activation and, intracellular ATP and GTP are involved in microglial mobility and secretory processes of inflammatory cytokines (101, 108–111).

While microglial activation is reported after irradiation in both juvenile and adult rodent brain (74, 112) intriguingly, we observed trivial Iba1^+^ cells in the radiation control, indicating a possible clearance of the activated microglia over time, as the brain samples were harvested 58 days post-IR, at a time-point close to moribund for tumor-bearing mice groups. The observed microglial morphology and tumor-stromal cross talk in tumor bearing mice implies that radiation induced metabolic alterations in brain stroma along with progressive pro-inflammatory damage caused by tumor growth could lead to continued feed-forward recruitment and activation of microglial cells at the tumor. Since, maximum deleterious alterations and tissue damage would be expected within 20 Gy radiated-GBM143 tumors, maximal phagocytic activity of microglia was observed in these, indicated by their all amoeboid phenotype.

The dose and time-dependence of radiation exposure can significantly alter the impact of RT on TME by affecting tumor or stromal cell behavior, migration, and treatment response (27, 29, 30, 113–120). The association between cancer, aging and therapy-associated aging is well documented (50, 51). High-dose IR effects include hemorrhage, cognitive decline, neurodegeneration, and premature senescence, which can progress over time (13, 15).

Multivariate analysis of ^1^H-NMR data and heatmap of GC-MS data revealed a clear distinction between aged-mice groups from 20 Gy-single dose (Figures S2B,Cii). The metabolic changes observed in aged and irradiated-mice differed markedly in relative abundance of most of the metabolites assessed by ^1^H-NMR and GC-MS (Figures 3, 4). Increased levels of urea and decreases in NAA and creatine (Cr) or Crn levels have been observed in neuropathologies (116, 121). We observed a slight increase in urea with radiation in both mouse strains, but NAA and Crn levels were not consistent and demonstrated a decline only observed in athymic nude mice. These indicate a partial neurotoxic state induced by 20 Gy-IR; with no severe aging-like signatures in 20 Gy. This could in part be due to the time-dependence of the experiment, where mice brain samples were harvested for metabolic analysis 24 hrs post-RT to mimic the time frame in which tumor implantations were performed post IR.

The association between the metabolic effects and time since radiation was investigated by performing a survival analysis. Shortest median survival in the LT-IR cohort indicates progressive IR-induced damage in tumor stroma, making it more permissive for tumor growth and recurrence. This corresponds to progressive radiation-induced brain injury and increased susceptibility to neuropathologies observed in patients treated with RT (122). Clinical correlation of the study is depicted in Figure S5.

## Conclusions

We identified an aggressive tumor behavior and microglial activation following 20 Gy single dose brain radiation, which could become more severe with time. Moreover, we found metabolic alterations with a rise in energy carriers (ATP and GTP) and a decline in antioxidants ASC and GSH to associate with the observed tumor phenotype. Independent groups have reported metabolic alterations in GBM cells to be pro-tumorigenic (21–25). We show for the first time a comprehensive view over the metabolomic alterations in the pre-radiated brain administered with high-dose IR (equivalent to late effects of hypo-fractionated dose), *in vivo*, that associate with tumor proliferation, migration, and inflammatory phenotype. These observations suggest an unprecedented role of the pre-radiated brain microenvironment on aggressive GBM recurrence, with, sustained and progressive metabolic stresses to worsen GBM outcome.

## Future Direction

The role of antioxidants in compromising the therapeutic effect of RT and pro-oxidants in sensitization to RT has long been debated (123–134). Radiation therapy mediates its effects directly or indirectly by production of ROS; thereby, causing oxidative damage to macromolecules and induction of apoptosis. Therefore, increased expression of antioxidant peptides in tumors have been thought to reduce the cytotoxic effects of RT, and GSH inhibition is proposed to have a therapeutic advantage in sensitizing cells to RT (73, 135). Ascorbate can act as a pro-oxidant in acidic microenvironments, such as tumors (136); thus, it may function as a radio-sensitizer for GBM cells and a radioprotector for normal cells post-RT (137, 138). While discrepancies remain regarding ASC's role as a radio-sensitizer or radio-protector in GBM, its potential as an anticancer agent has been reviewed (139–143).

Our study demonstrates an immediate effect of prior exposure to high-dose irradiation in the non-tumor/untransformed brain cells as a decrease in antioxidant levels, including GSH and ASC, consistent with their utilization to neutralize RT-induced free radicals. The depletion of these antioxidants can lead to further acute or chronic oxidative stress, altering the brain TME, which may contribute to the enhanced aggressiveness of recurrent tumors. While radiation-induced oxidative stress is necessary for DNA damage in tumor cells, this study raises the question if GSH and ASC administration after completion of radiation or primary treatment regime could help mitigate the radiation-induced metabolic stress in the microenvironment. If the post-radiation redox state contributes to tumor aggressiveness, there may be an opportunity to attenuate the RT-associated aggressiveness of recurrent GBM, enhancing the long-term safety of brain radiation treatment for glioblastoma (translational relevance illustrated in Figure S5).

## Data Availability Statement

All datasets generated for this study are included in the article/Supplementary Material.

## Ethics Statement

The animal study was reviewed and approved by the Institutional Animal Care and Use Committee (IACUC) at Mayo Clinic, Rochester.

## Author Contributions

KG and TB led the project, contributed to experimental design, review, and discussion. TB supervised and supported KG. KG, YX, and BC carried-out mice tumor experiments. KG conducted survival studies and performed immunostainings. BC supervised KG on irradiator operation. IO assisted KG. KG and JJ collaborated to analyze images. SM and IV conceived ^1^H-NMR protocol. KG, IV, and SZ performed ^1^H-NMR and GC-MS studies, and data analysis. Metabolomics core provided support with LC-MS and data analysis. All authors contributed to experiments and research execution. Figures provided by KG and IV. Illustrations created by KG. All authors contributed to manuscript writing, research, editing, and final review.

## Conflict of Interest

The authors declare that the research was conducted in the absence of any commercial or financial relationships that could be construed as a potential conflict of interest.

## Supplementary Material

Figure S1 Tumor growth assessed post-moribund for cranially irradiated mice **(A–E)**: **(A)** GBM143 PDX line obtained from flank tumor, cultured *in vitro* for 3 weeks. Images acquired in three independent fields (F1–F3), using transmitted light microscopy (at 10X) indicate morphology of cells to be branched, neuroglia-like, interspersed with enlarged polygonal cells. **(B)** Scheme for the slicing strategy: Mice brain was sectioned into four equidistant pieces (~1.8 mm apart); Slices were made from each in the order of being 5 μm thick coronal slices from the cerebral hemisphere only, Rostral to caudal for 22 slides, so as to cover a depth of 120 μm from each of the four tissue pieces. These slices were arranged onto the glass slides, such that each slice on a slide is obtained from one of the respective four brain pieces, sectioned equidistantly. Two slides (1 and 22) were stained with H&E and evaluated for tumor growth. Tumor positive area was detected in slices obtained from two out of four sectioned pieces for most of the mice brain samples. Percent positive H and E staining was assessed for each. **(C)** Illustration showing arrangement of the slices on a glass slide, and evaluation of percent positive H&E. **(D)** Relative H&E staining as observed for slices obtained from 0 Gy, 10 Gy, and 20 Gy. Dot-plot for the overall tumor burden estimated in these groups. **(E)** Scheme illustrating steps involved in performing single cell count: mice brain coronal sections are stained for h-Lamin A+C –Cy3 (and Ki67–Cy5), for both 0 Gy and 20 Gy. A defined region is selected and masked (area-squared in white). This masked area-image in single channels is imported into cell profiler software and cropped. This cropped image is used as the input image, pipeline for nuclei detection run, and single cell count obtained. Similar steps are performed for a defined region selected at center of corpus callosum for h-LaminA+C staining (images in box, on right). **(F)** Effects of radiation induced alterations on GBM outcome: Scheme of experiment for survival analysis in athymic nude mice groups, 0 Gy and 20 Gy irradiated.

Figure S2 **(A)** Scheme for experiment involving Proton–Nuclear Magnetic Resonance spectroscopy (^1^H-NMR) and Gas chromatography–mass spectrometry (GC-MS). **(B)** ^1^H-NMR: Multivariate analysis for C57BL/6 mice, having groups as indicated. Supervised Orthogonal Partial Least Square-Discriminate Analysis (OPLS-DA) to show further separation of 0 Gy, with irradiated group, irradiation (IR) (20 and 4 Gy × 10); (i) Total variable importance in the projection (VIP) values (ii) Predicted VIP values. Parameters involved in group separation using multivariate analysis in M1–M7 models are listed in the Table S1. **(C)** Heatmaps for GC-MS data: (i) Heatmap for relative abundance of metabolites (i.e., normalized total peak area of metabolites for all mice within each group) between athymic nude mice groups, 0 Gy and 20 Gy. (ii) Heatmap for relative abundance of metabolites averaged for each group (i.e., normalized total peak area for metabolites, averaged for all mice within each group), between C57BL/6 mice grouped indicated.

Figure S3 Proton–Nuclear Magnetic resonance spectroscopy (^1^H-NMR): The graphs show, relative abundance of metabolites between C57BL/6 mice groups. Group Comparison: Aged (24 mo), Aged-Obese (24 mo) verses control (0 Gy), & radiated (20 Gy, 4 Gy × 10). The significantly altered metabolites are categorized as per their molecular type or biological pathway involvement. Statistical significance is represented as ^*^*p* < 0.05; ^**^*p* < 0.01; ^***^*p* < 0.001, ^****^*p* < 0.0001.

Figure S4 Gas chromatography–mass spectrometry (GC-MS): Group Comparison: Aged (24 mo), Aged-Obese (24 mo) verses control (0 Gy), and radiated (20 Gy, 4 Gy × 10). The graphs show, relative abundance of metabolites between C57BL/6 mice groups. The significantly altered metabolites are categorized as per their molecular type or biological pathway involvement. Statistical significance is represented as ^*^*p* < 0.05; ^**^*p* < 0.01; ^***^*p* < 0.001, ^****^*p* < 0.0001.

Figure S5 Translational Neuro-Oncology: The model illustrates sequential alterations that may contribute to tumor recurrence post-primary treatment regime. Standard of care for glioblastoma multiforme (GBM) involves tumor resection, radiation therapy (RT) and chemotherapy (Temozolomide, TMZ). Residual tumor cells or glioblastoma stem cells (after primary treatment regime) have the ability to migrate away from initial site, if their surrounding microenvironment becomes liberal for it. Radiation induced alterations in brain parenchyma and its extracellular microenvironment (or tumor stromal compartment), include metabolic changes such as reduced antioxidants, increase in energy carriers, neuroinflamation, and others. These changes can dramatically remodel the pre-radiated brain stroma, making it permissive for tumor cells to re-grow and migrate to distant sites forming new foci; thereby, causing tumor recurrence and spread. Future therapeutic interventions to prevent secondary tumor growth may harness these insights to leverage the potential of radiation therapy, and better treatment with use of cell proliferation and migration inhibitors and metabolic modulators to advance GBM care.

Table S1 Model parameters used for multivariate analysis of ^1^H-NMR data.

## Abbreviations

RT: Radiation therapy
IR: Irradiation
GBM: Glioblastoma
PDX: Patient derived xenograft
IC: Intracranial
TME: Tumor microenvironment
IH: Ipsilateral hemisphere
CH: Contralateral hemisphere
TAM: Tumor associated macrophages
CBCT: Cone beam computed tomography
IF: Immunofluorescence
H&E: Hematoxylin and Eosin
^1^H-NMR: Proton–Nuclear Magnetic Resonance spectroscopy
GC-MS: Gas chromatography–mass spectrometry
LC-MS: Liquid chromatography–mass spectrometry
PCA: Principle Component Analysis
PLS-DA: Partial least squares–Discriminant Analysis
OPLS-DA: Orthogonal Partial least squares–Discriminant Analysis
VIP: Variable Importance on Projection
VIP-pred: Value of the VIP variant for the predictive components
VIP-total: Total sum of VIP values for both predictive and orthogonal components
ROS: Reactive oxygen species
eROS: Extracellular reactive oxygen species
RNS: Reactive nitrogen species
TCA: Tricarboxylic Acid cycle
NAD: Nicotinamide adenine dinucleotide
ATP: Adenosine triphosphate
GTP: Guanosine triphosphate
eATP/eGTP: Extracellular ATP/Extracellular GTP
GSH: Glutathione (reduced)
ASC: Ascorbate
ThrO: Threonic acid
OxA: Oxalic acid
NAA: N-acetyl aspartate
Crn: Creatinine
Cr: Creatine
PC: Phosphocholine
Ob (Aged Ob): Obese (Aged Obese)
Gy: Gray
hrs/mo: Hours/Months.
