# Supplementary material for: Radiation Induced Metabolic Alterations Associate With Tumor Aggressiveness and Poor Outcome in Glioblastoma
Source: Front Oncol. 2020 May 5;10:535. doi: 10.3389/fonc.2020.00535 (PMC7214818; doi:10.3389/fonc.2020.00535)
Supplement: Figure S1 — Tumor growth assessed post-moribund for cranially irradiated mice (A–E): (A) GBM143 PDX line obtained from flank tumor, cultured in vitro for 3 weeks. Images acquired in three independent fields (F1–F3), using transmitted light microscopy (at 10X) indicate morphology of cells to be branched, neuroglia-like, interspersed with enlarged polygonal cells. (B) Scheme for the slicing strategy: Mice brain was sectioned into four equidistant pieces (~1.8 mm apart); Slices were made from each in the order of being 5 μm thick coronal slices from the cerebral hemisphere only, Rostral to caudal for 22 slides, so as to cover a depth of 120 μm from each of the four tissue pieces. These slices were arranged onto the glass slides, such that each slice on a slide is obtained from one of the respective four brain pieces, sectioned equidistantly. Two slides (1 and 22) were stained with H&E and evaluated for tumor growth. Tumor positive area was detected in slices obtained from two out of four sectioned pieces for most of the mice brain samples. Percent positive H and E staining was assessed for each. (C) Illustration showing arrangement of the slices on a glass slide, and evaluation of percent positive H&E. (D) Relative H&E staining as observed for slices obtained from 0 Gy, 10 Gy, and 20 Gy. Dot-plot for the overall tumor burden estimated in these groups. (E) Scheme illustrating steps involved in performing single cell count: mice brain coronal sections are stained for h-Lamin A+C –Cy3 (and Ki67–Cy5), for both 0 Gy and 20 Gy. A defined region is selected and masked (area-squared in white). This masked area-image in single channels is imported into cell profiler software and cropped. This cropped image is used as the input image, pipeline for nuclei detection run, and single cell count obtained. Similar steps are performed for a defined region selected at center of corpus callosum for h-LaminA+C staining (images in box, on right). (F) Effects of radiation induced alterations on GBM out [file Presentation_1.pptx]

## Slide 1
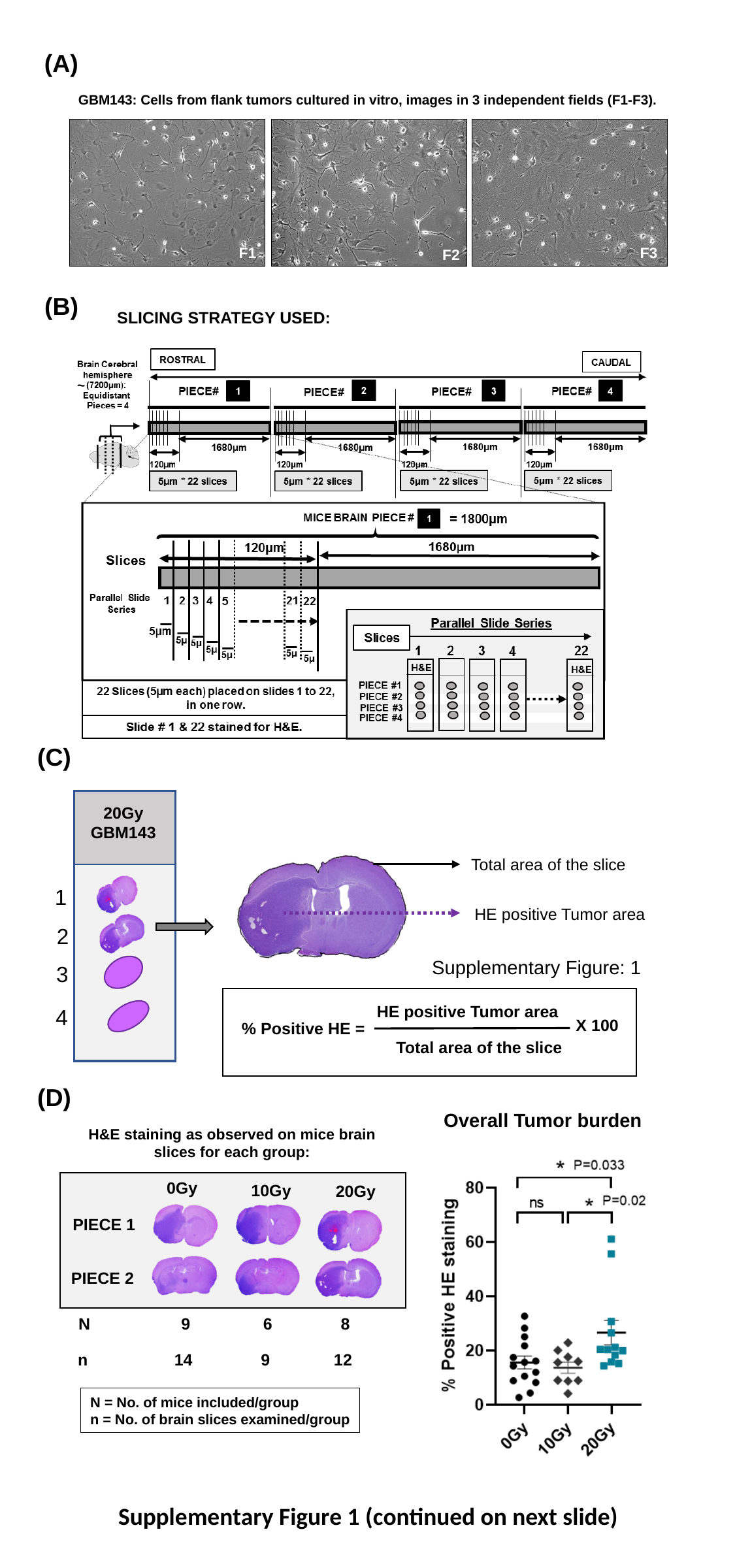

(A)
GBM143: Cells from flank tumors cultured in vitro, images in 3 independent fields (F1-F3).
F3
F1
F2
(B)
SLICING STRATEGY USED:
~
(C)
20Gy
GBM143
Total area of the slice
1
HE positive Tumor area
2
Supplementary Figure: 1
3
HE positive Tumor area
X 100
% Positive HE =
Total area of the slice
4
(D)
Overall Tumor burden
H&E staining as observed on mice brain slices for each group:
0Gy
10Gy
20Gy
PIECE 1
PIECE 2
N 9 6 8
n 14 9 12
N = No. of mice included/group
n = No. of brain slices examined/group
Supplementary Figure 1 (continued on next slide)

## Slide 2
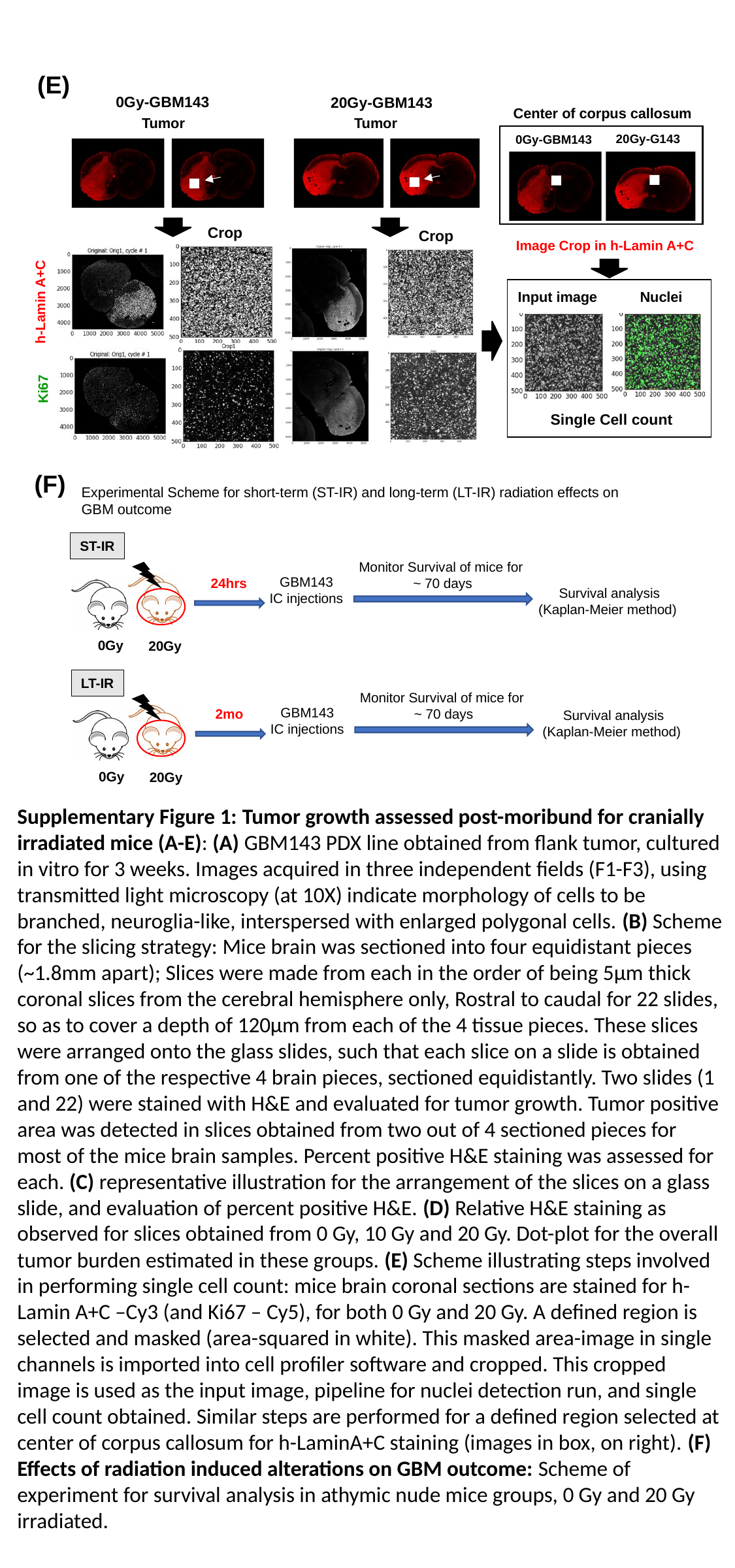

(E)
0Gy-GBM143
Crop
20Gy-GBM143
Crop
Center of corpus callosum
Tumor
Tumor
20Gy-G143
0Gy-GBM143
Image Crop in h-Lamin A+C
Input image
Nuclei
Single Cell count
h-Lamin A+C
Ki67
(F)
Experimental Scheme for short-term (ST-IR) and long-term (LT-IR) radiation effects on GBM outcome
ST-IR
Monitor Survival of mice for
~ 70 days
0Gy
20Gy
GBM143
IC injections
24hrs
Survival analysis
(Kaplan-Meier method)
LT-IR
Monitor Survival of mice for
~ 70 days
0Gy
20Gy
GBM143
IC injections
2mo
Survival analysis
(Kaplan-Meier method)
Supplementary Figure 1: Tumor growth assessed post-moribund for cranially irradiated mice (A-E): (A) GBM143 PDX line obtained from flank tumor, cultured in vitro for 3 weeks. Images acquired in three independent fields (F1-F3), using transmitted light microscopy (at 10X) indicate morphology of cells to be branched, neuroglia-like, interspersed with enlarged polygonal cells. (B) Scheme for the slicing strategy: Mice brain was sectioned into four equidistant pieces (~1.8mm apart); Slices were made from each in the order of being 5μm thick coronal slices from the cerebral hemisphere only, Rostral to caudal for 22 slides, so as to cover a depth of 120μm from each of the 4 tissue pieces. These slices were arranged onto the glass slides, such that each slice on a slide is obtained from one of the respective 4 brain pieces, sectioned equidistantly. Two slides (1 and 22) were stained with H&E and evaluated for tumor growth. Tumor positive area was detected in slices obtained from two out of 4 sectioned pieces for most of the mice brain samples. Percent positive H&E staining was assessed for each. (C) representative illustration for the arrangement of the slices on a glass slide, and evaluation of percent positive H&E. (D) Relative H&E staining as observed for slices obtained from 0 Gy, 10 Gy and 20 Gy. Dot-plot for the overall tumor burden estimated in these groups. (E) Scheme illustrating steps involved in performing single cell count: mice brain coronal sections are stained for h-Lamin A+C –Cy3 (and Ki67 – Cy5), for both 0 Gy and 20 Gy. A defined region is selected and masked (area-squared in white). This masked area-image in single channels is imported into cell profiler software and cropped. This cropped image is used as the input image, pipeline for nuclei detection run, and single cell count obtained. Similar steps are performed for a defined region selected at center of corpus callosum for h-LaminA+C staining (images in box, on right). (F) Effects of radiation induced alterations on GBM outcome: Scheme of experiment for survival analysis in athymic nude mice groups, 0 Gy and 20 Gy irradiated.

## Slide 3
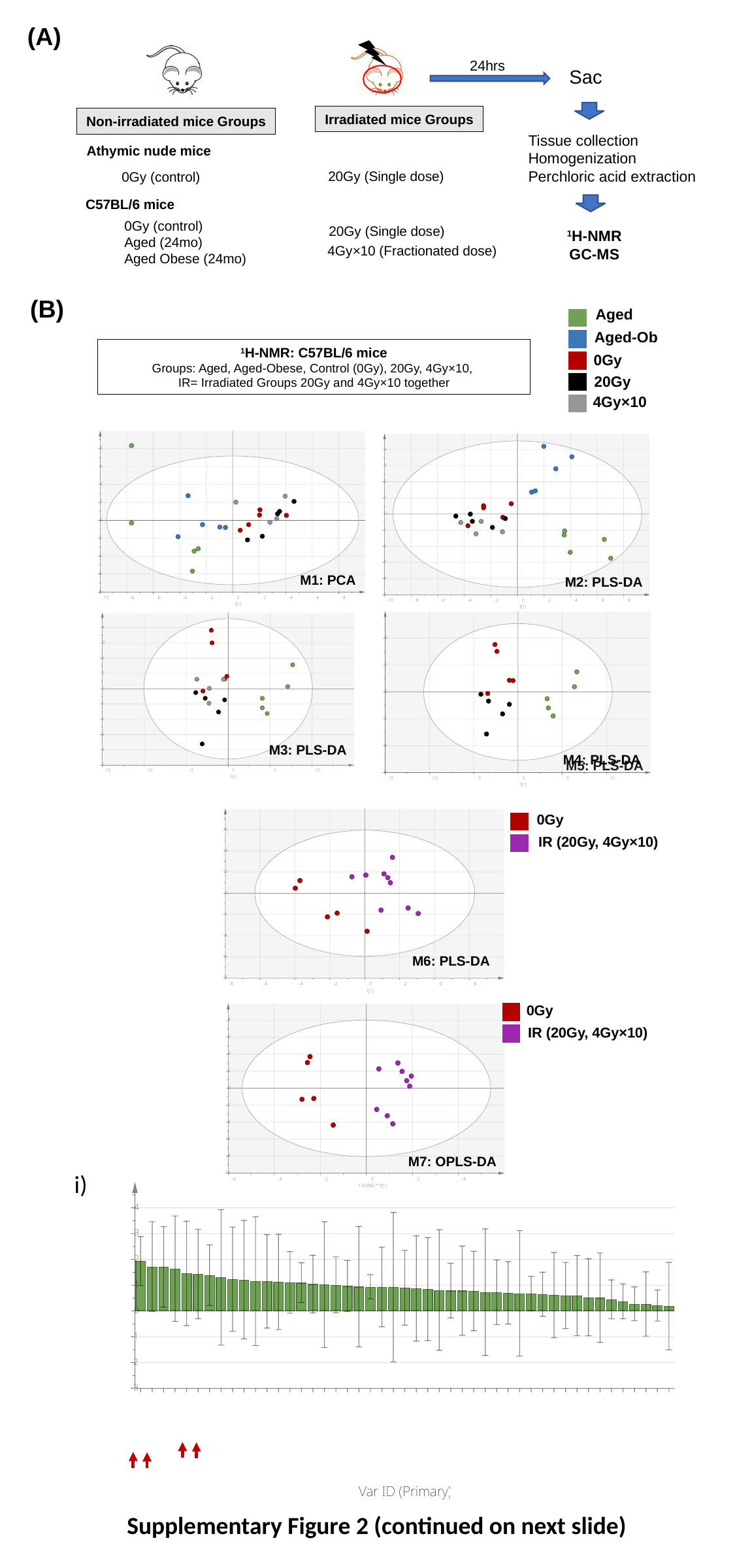

(A)
24hrs
Sac
Tissue collection
Homogenization
Perchloric acid extraction
Irradiated mice Groups
Non-irradiated mice Groups
Athymic nude mice
20Gy (Single dose)
0Gy (control)
C57BL/6 mice
0Gy (control)
Aged (24mo)
Aged Obese (24mo)
20Gy (Single dose)
4Gy×10 (Fractionated dose)
1H-NMR
GC-MS
(B)
Aged
Aged-Ob
0Gy
20Gy
4Gy×10
1H-NMR: C57BL/6 mice
Groups: Aged, Aged-Obese, Control (0Gy), 20Gy, 4Gy×10,
IR= Irradiated Groups 20Gy and 4Gy×10 together
M2: PLS-DA
M1: PCA
M4: PLS-DA
M3: PLS-DA
M5: PLS-DA
M6: PLS-DA
0Gy
IR (20Gy, 4Gy×10)
0Gy
M7: OPLS-DA
IR (20Gy, 4Gy×10)
i)
Supplementary Figure 2 (continued on next slide)

## Slide 4
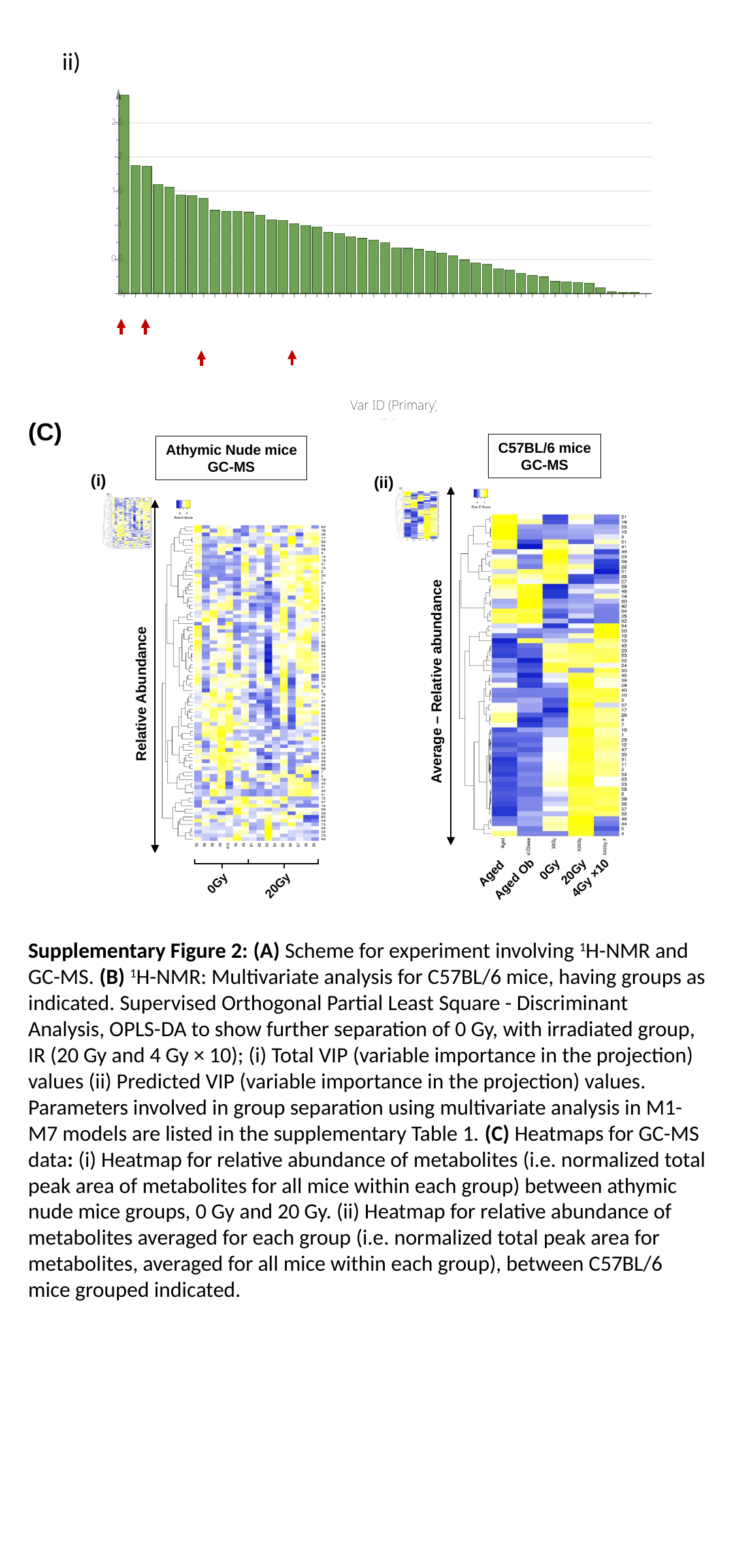

ii)
(C)
C57BL/6 mice
GC-MS
Athymic Nude mice
GC-MS
(i)
(ii)
 Average – Relative abundance
0Gy
4Gy ×10
20Gy
Aged
Aged Ob
Relative Abundance
0Gy
20Gy
Supplementary Figure 2: (A) Scheme for experiment involving 1H-NMR and GC-MS. (B) 1H-NMR: Multivariate analysis for C57BL/6 mice, having groups as indicated. Supervised Orthogonal Partial Least Square - Discriminant Analysis, OPLS-DA to show further separation of 0 Gy, with irradiated group, IR (20 Gy and 4 Gy × 10); (i) Total VIP (variable importance in the projection) values (ii) Predicted VIP (variable importance in the projection) values. Parameters involved in group separation using multivariate analysis in M1-M7 models are listed in the supplementary Table 1. (C) Heatmaps for GC-MS data: (i) Heatmap for relative abundance of metabolites (i.e. normalized total peak area of metabolites for all mice within each group) between athymic nude mice groups, 0 Gy and 20 Gy. (ii) Heatmap for relative abundance of metabolites averaged for each group (i.e. normalized total peak area for metabolites, averaged for all mice within each group), between C57BL/6 mice grouped indicated.

## Slide 5
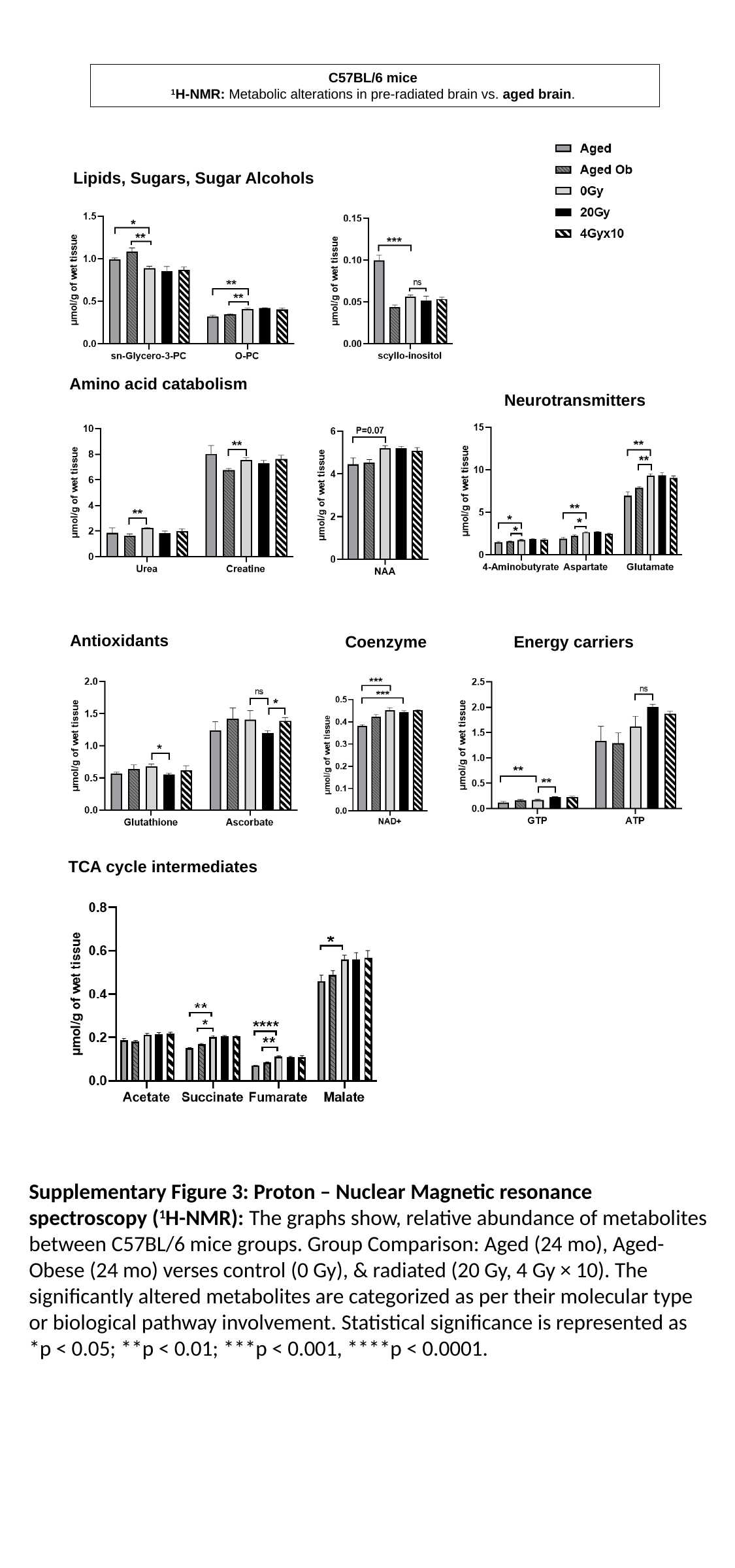

C57BL/6 mice
1H-NMR: Metabolic alterations in pre-radiated brain vs. aged brain.
Lipids, Sugars, Sugar Alcohols
Amino acid catabolism
Neurotransmitters
Antioxidants
Energy carriers
Coenzyme
TCA cycle intermediates
Supplementary Figure 3: Proton – Nuclear Magnetic resonance spectroscopy (1H-NMR): The graphs show, relative abundance of metabolites between C57BL/6 mice groups. Group Comparison: Aged (24 mo), Aged-Obese (24 mo) verses control (0 Gy), & radiated (20 Gy, 4 Gy × 10). The significantly altered metabolites are categorized as per their molecular type or biological pathway involvement. Statistical significance is represented as *p < 0.05; **p < 0.01; ***p < 0.001, ****p < 0.0001.

## Slide 6
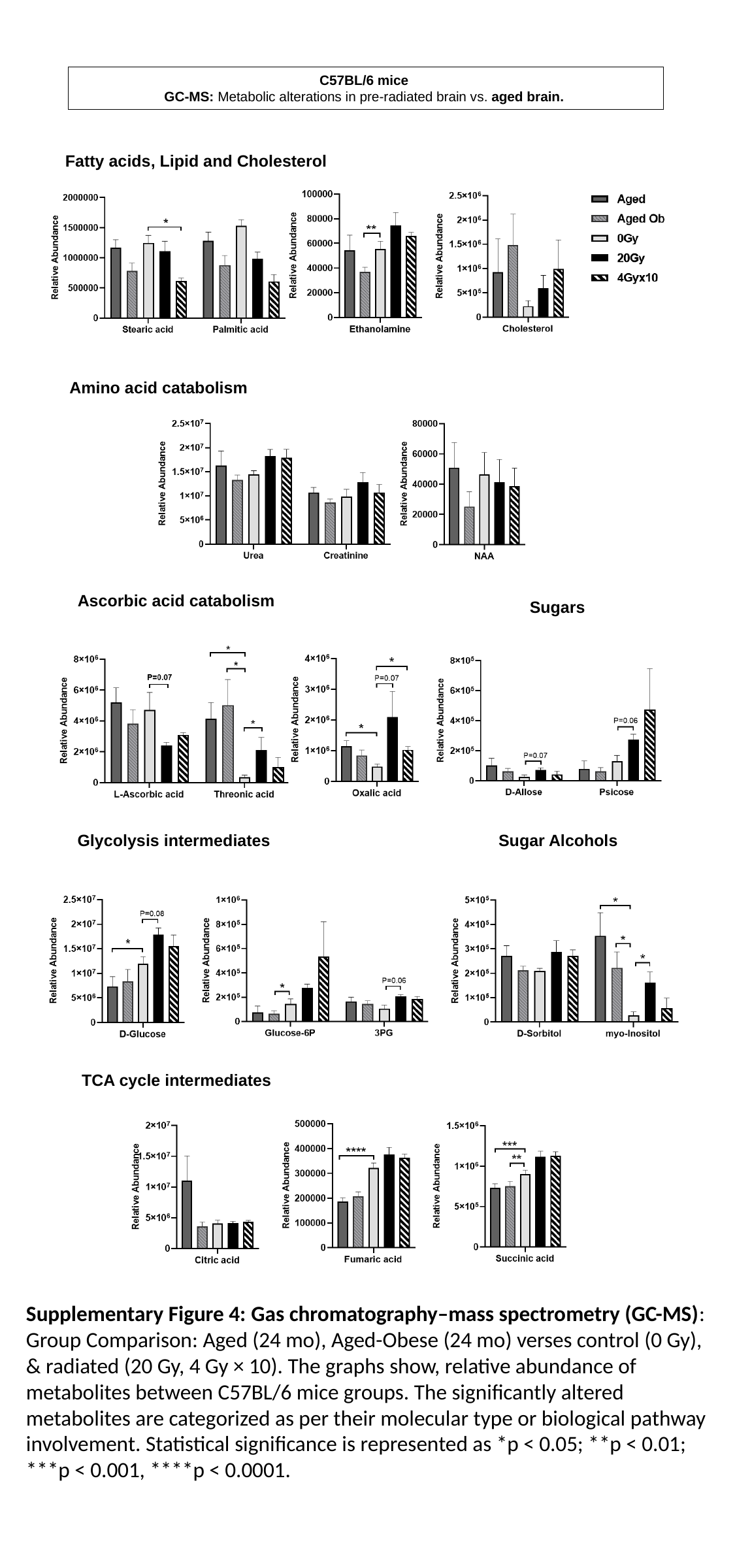

C57BL/6 mice
GC-MS: Metabolic alterations in pre-radiated brain vs. aged brain.
Fatty acids, Lipid and Cholesterol
Amino acid catabolism
Ascorbic acid catabolism
Sugars
Glycolysis intermediates
Sugar Alcohols
TCA cycle intermediates
Supplementary Figure 4: Gas chromatography–mass spectrometry (GC-MS): Group Comparison: Aged (24 mo), Aged-Obese (24 mo) verses control (0 Gy), & radiated (20 Gy, 4 Gy × 10). The graphs show, relative abundance of metabolites between C57BL/6 mice groups. The significantly altered metabolites are categorized as per their molecular type or biological pathway involvement. Statistical significance is represented as *p < 0.05; **p < 0.01; ***p < 0.001, ****p < 0.0001.

## Slide 7
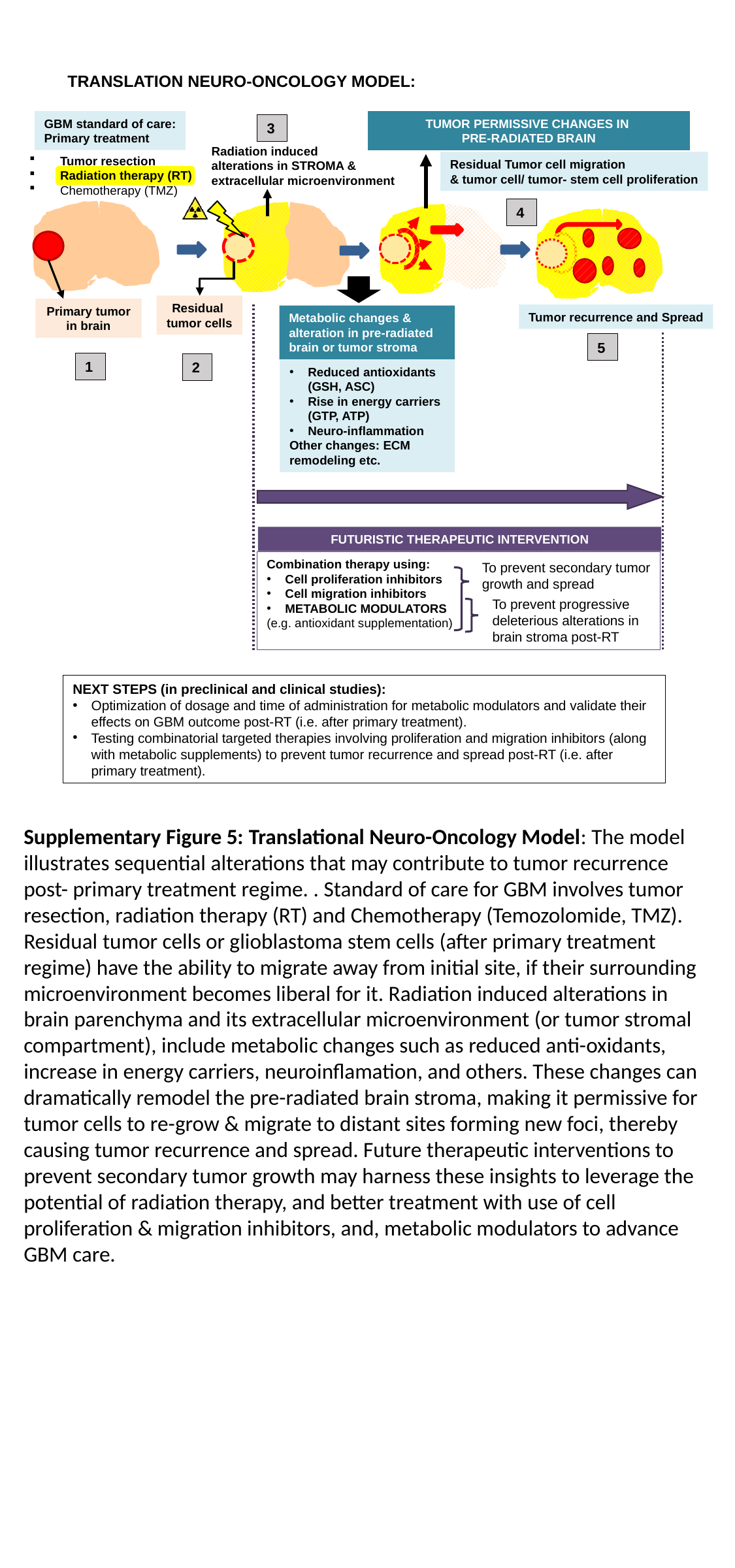

TRANSLATION NEURO-ONCOLOGY MODEL:
GBM standard of care:
Primary treatment
TUMOR PERMISSIVE CHANGES IN
PRE-RADIATED BRAIN
3
Radiation induced
alterations in STROMA &
extracellular microenvironment
Tumor resection
Radiation therapy (RT)
Chemotherapy (TMZ)
Residual Tumor cell migration
& tumor cell/ tumor- stem cell proliferation
4
Residual
tumor cells
Primary tumor
in brain
Tumor recurrence and Spread
Metabolic changes & alteration in pre-radiated brain or tumor stroma
Reduced antioxidants (GSH, ASC)
Rise in energy carriers (GTP, ATP)
Neuro-inflammation
Other changes: ECM remodeling etc.
5
1
2
FUTURISTIC THERAPEUTIC INTERVENTION
Combination therapy using:
Cell proliferation inhibitors
Cell migration inhibitors
METABOLIC MODULATORS
(e.g. antioxidant supplementation)
To prevent secondary tumor growth and spread
To prevent progressive deleterious alterations in brain stroma post-RT
NEXT STEPS (in preclinical and clinical studies):
Optimization of dosage and time of administration for metabolic modulators and validate their effects on GBM outcome post-RT (i.e. after primary treatment).
Testing combinatorial targeted therapies involving proliferation and migration inhibitors (along with metabolic supplements) to prevent tumor recurrence and spread post-RT (i.e. after primary treatment).
Supplementary Figure 5: Translational Neuro-Oncology Model: The model illustrates sequential alterations that may contribute to tumor recurrence post- primary treatment regime. . Standard of care for GBM involves tumor resection, radiation therapy (RT) and Chemotherapy (Temozolomide, TMZ). Residual tumor cells or glioblastoma stem cells (after primary treatment regime) have the ability to migrate away from initial site, if their surrounding microenvironment becomes liberal for it. Radiation induced alterations in brain parenchyma and its extracellular microenvironment (or tumor stromal compartment), include metabolic changes such as reduced anti-oxidants, increase in energy carriers, neuroinflamation, and others. These changes can dramatically remodel the pre-radiated brain stroma, making it permissive for tumor cells to re-grow & migrate to distant sites forming new foci, thereby causing tumor recurrence and spread. Future therapeutic interventions to prevent secondary tumor growth may harness these insights to leverage the potential of radiation therapy, and better treatment with use of cell proliferation & migration inhibitors, and, metabolic modulators to advance GBM care.

## Slide 8
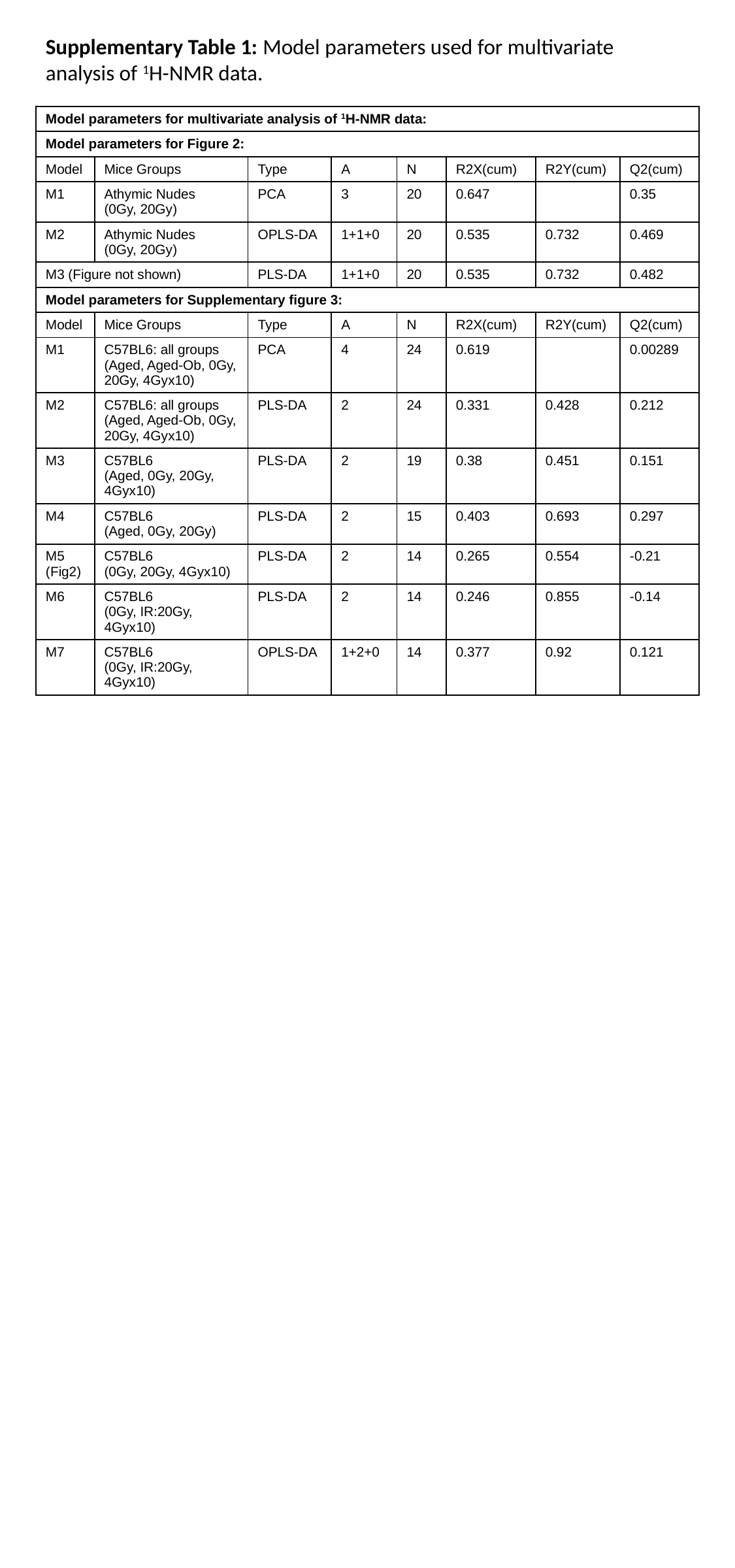

Supplementary Table 1: Model parameters used for multivariate analysis of 1H-NMR data.
| Model parameters for multivariate analysis of 1H-NMR data: | | | | | | | |
| --- | --- | --- | --- | --- | --- | --- | --- |
| Model parameters for Figure 2: | | | | | | | |
| Model | Mice Groups | Type | A | N | R2X(cum) | R2Y(cum) | Q2(cum) |
| M1 | Athymic Nudes (0Gy, 20Gy) | PCA | 3 | 20 | 0.647 | | 0.35 |
| M2 | Athymic Nudes (0Gy, 20Gy) | OPLS-DA | 1+1+0 | 20 | 0.535 | 0.732 | 0.469 |
| M3 (Figure not shown) | | PLS-DA | 1+1+0 | 20 | 0.535 | 0.732 | 0.482 |
| Model parameters for Supplementary figure 3: | | | | | | | |
| Model | Mice Groups | Type | A | N | R2X(cum) | R2Y(cum) | Q2(cum) |
| M1 | C57BL6: all groups (Aged, Aged-Ob, 0Gy, 20Gy, 4Gyx10) | PCA | 4 | 24 | 0.619 | | 0.00289 |
| M2 | C57BL6: all groups (Aged, Aged-Ob, 0Gy, 20Gy, 4Gyx10) | PLS-DA | 2 | 24 | 0.331 | 0.428 | 0.212 |
| M3 | C57BL6 (Aged, 0Gy, 20Gy, 4Gyx10) | PLS-DA | 2 | 19 | 0.38 | 0.451 | 0.151 |
| M4 | C57BL6 (Aged, 0Gy, 20Gy) | PLS-DA | 2 | 15 | 0.403 | 0.693 | 0.297 |
| M5 (Fig2) | C57BL6 (0Gy, 20Gy, 4Gyx10) | PLS-DA | 2 | 14 | 0.265 | 0.554 | -0.21 |
| M6 | C57BL6 (0Gy, IR:20Gy, 4Gyx10) | PLS-DA | 2 | 14 | 0.246 | 0.855 | -0.14 |
| M7 | C57BL6 (0Gy, IR:20Gy, 4Gyx10) | OPLS-DA | 1+2+0 | 14 | 0.377 | 0.92 | 0.121 |
